# Effective data sampling strategies and boundary condition constraints of physics-informed neural networks for identifying material properties in solid mechanics

**DOI:** 10.1007/s10483-023-2995-8

**Published:** 2023-07-03

**Authors:** W. WU, M. DANEKER, M. A. JOLLEY, K. T. TURNER, L. LU

**Affiliations:** 1. Department of Anesthesiology and Critical Care Medicine, Children’s Hospital of Philadelphia, Philadelphia, PA 19104, U. S. A.; 2. Division of Pediatric Cardiology, Children’s Hospital of Philadelphia, Philadelphia, PA 19104, U. S. A.; 3. Department of Chemical and Biomolecular Engineering, University of Pennsylvania, Philadelphia, PA 19104, U. S. A.; 4. Department of Mechanical Engineering and Applied Mechanics, University of Pennsylvania, Philadelphia, PA 19104, U. S. A.

**Keywords:** solid mechanics, material identification, physics-informed neural network (PINN), data sampling, boundary condition (BC) constraint, O242, O343, 35Q74, 65M32, 74B20, 74G75

## Abstract

Material identification is critical for understanding the relationship between mechanical properties and the associated mechanical functions. However, material identification is a challenging task, especially when the characteristic of the material is highly nonlinear in nature, as is common in biological tissue. In this work, we identify unknown material properties in continuum solid mechanics via physics-informed neural networks (PINNs). To improve the accuracy and efficiency of PINNs, we develop efficient strategies to nonuniformly sample observational data. We also investigate different approaches to enforce Dirichlet-type boundary conditions (BCs) as soft or hard constraints. Finally, we apply the proposed methods to a diverse set of time-dependent and time-independent solid mechanic examples that span linear elastic and hyperelastic material space. The estimated material parameters achieve relative errors of less than 1%. As such, this work is relevant to diverse applications, including optimizing structural integrity and developing novel materials.

## 1 Introduction

The study of nonlinear dynamical systems is of interest in many science and engineering disciplines due to the nonlinear nature of most physical phenomena. The nonlinear relation between system inputs and outputs and interactions between system variables have made nonlinear systems a daunting task to solve with traditional analytical approaches. In engineering, the solutions to nonlinear dynamical systems are approximated by classic numerical methods (i.e., the finite difference method, the finite element method, or the finite volume method). These numerical methods discretize a large system into finite spatial subcomponents, linearize the governing equations in time, and solve the linearized equations iteratively until each subcomponent satisfies the governing conservation laws. However, this spatio-temporal discretization procedure often introduces spurious oscillation and requires numerical damping in order to obtain stable solutions, which may lead to less accurate approximations^[1-4]^. On the contrary, the universal approximation theorem of neural networks^[5]^ states that a sufficiently large feed-forward artificial neural network with proper nonlinear activation functions can approximate any continuous function. As such, machine learning-based approaches have arisen as a new paradigm for addressing physical problems that are known to be challenging for classic numerical methods^[6]^. These recent advances in deep learning techniques have demonstrated considerable potential for unveiling the hidden physics of many complex nonlinear dynamical systems where classic numerical methods fail^[6-10]^.

The tremendous growth of deep learning has also attracted material scientists’ attention to accelerating the understanding of complex material properties. A comprehensive understanding of mechanical properties is essential for studying the behaviors of materials under loads. In the traditional engineering approach, the material investigation (material parameter estimation) procedure is generally carried out in the following three steps: (i) conduct a series of experimental studies to quantify the mechanical behavior of the material; (ii) identify a representative mathematical model by performing a series of inverse finite element analyses (FEAs); (iii) use optimization techniques to identify the unknown parameters in the mathematical model that produce the best agreement with experimental results. However, the inversion process can be computationally expensive, or even impossible, for complex and nonlinear models owing to a large number of forward simulations required^[11]^. As a result, data-driven deep learning has been leveraged as a surrogate modeling technique to study the nonlinear deformation relationship between material behavior and load conditions^[12-17]^, as well as to design new materials with unique mechanical characteristics^[18-23]^.

Despite the efficacy of deep learning in interpreting complex systems, this promising method is not without shortcomings. First, the accuracy of deep learning prediction is highly reliant on the volume of data^[24]^. Second, conventional neural networks trained purely on data are unrestricted to the system’s underlying governing equations and boundary conditions (BCs); this limits the capability to extrapolate accurate physical relations from network outputs beyond their training data^[13]^. As a solution to this limitation, physics-informed neural networks (PINNs)^[25-26]^ have emerged to improve the training process by integrating mathematical models. PINNs use neural networks to approximate the solution and encode the governing equations (e.g., the ordinary differential equations (ODEs) or partial differential equations (PDEs)) in the loss function. For an inverse problem, this loss function encompasses the residual of the initial conditions (ICs), the BCs, the PDE at specific collocation points in the physical domain, and observation data. Incorporating physical laws ensures that the networks satisfy both phenomenological observations from data and the underlying physical laws and constraints within the system, and therefore significantly improves the effectiveness of applying the trained models to unexplored data sets and for sensitivity analysis^[27]^. For example, Lu et al.^[14]^ demonstrated that integrating physical laws and experimental data results in significantly improved solution accuracy for extracting material properties from instrumented indentation tests.

PINNs have succeeded in estimating the solutions to a wide range of forward and inverse problems, including classic differential problems^[9,26,28-33]^, fractional equations^[34]^, integral-differential equations^[25,35]^, and stochastic PDEs^[36]^. In recent years, researchers have used PINNs to address nonlinear solid mechanic problems by modifying the network architecture, the loss function expression^[37]^, and collocation point sampling methods. Samaniego et al.^[38]^, Nguyen-Thanh et al.^[39-40]^, and Abueidda et al.^[41]^ developed a deep energy method (i.e., the loss term for the PDEs was expressed in terms of the potential energy rather than the conservation of momentum) and demonstrated its applicability to steady-state elasticity, hyper-elasticity, viscoelasticity, and piezoelectricity problems. Fuhg and Bouklas^[42]^ discovered that incorporating stresses as additional outputs in the network enhances the network’s capability of resolving localized features in the solution for linear elastic steady-state problems. Haghighat et al.^[27]^ presented a parallel neural network architecture to identify material parameters for linear elastic and nonlinear-elastoplastic test problems using a pre-trained network. Henkes et al.^[43]^ presented an adaptive collocation sampling point framework to capture the underlying physics of microstructural elastostatics. Wu et al.^[44]^ proposed residual-based adaptive sampling methods and demonstrated significantly improved prediction accuracy for both forward and inverse problems. Rao et al.^[45]^ presented a PINN framework for solving forward elastic and elastodynamic problems with strongly enforced ICs and BCs. Zhang et al.^[46]^ presented a PINN framework to identify unknown geometric and material parameters of steady-state linear elastic, hyperelastic, and plastic materials with a pre-trained network.

In the present work, we aim to derive unknown material parameters in continuum solid mechanics. We focus on the application of PINNs to identify the unknown material parameters on higher-order initial-value and boundary-value problems. We identify the performance of enforcing Dirichlet-type BCs as soft or hard constraints. We also show the efficacy of several observation point sampling strategies on the two-dimensional (2D) examples for estimating linear elastic and hyperelastic materials. For the static problems, we consider the following three types: (i) concentrated data sampling in the location with high stress differential, (ii) uniform data sampling across the spatial domain, (iii) data sampling along the boundary only. For the dynamic example, we experiment with reducing the number of time frames in the reference data. We summarize our major contributions as follows.

(I) While there are an increasing number of works demonstrating that PINNs with hard constraints yield superior solution accuracy for forward problems^[43,47-48]^, the performance of hard constraints in inverse problems has not been systematically studied. In fact, soft constraints are more common in prior studies when solving inverse problems in solid mechanics^[27,46]^. We have presented first-of-its-kind comparative studies to investigate the effects of soft and hard constraints on a variety of inverse problems in solid mechanics. We have also demonstrated the importance of hard constraint auxiliary functions on solution accuracy.

(II) The point sampling strategies in the literature focus on the sampling of PDE residual points^[43,49]^. However, the ideal locations for selecting PDE residual points and collecting experimental data may not be the same. As such, identifying an optimal strategy for sampling observation data points is important in inverse problems. We examine the effect of observation (e.g., experimental) data sampling strategies on solution accuracy.

(III) In order to achieve satisfactory solution accuracy in PINNs, the governing PDEs often require non-dimensionalization such that the network outputs are in O(1). In this study, we propose an alternative technique using output transformation functions to map the output variables to their physical quantities. This method simplifies the PDE formulation and bypasses the need for non-dimensionalizing the PDEs.

Applying PINNs to inverse problems allows the discovery of material constitutive properties in a wide range of engineering domains when they are difficult or impossible to obtain otherwise. This work demonstrates a proof of concept of applying PINNs to uncover unknown material constants in test examples under various material types and loading conditions. We demonstrate the applicability of our PINN inversion framework to both steady-state and dynamic solid mechanics examples by applying our methods to the wave equation, Euler-Bernoulli beam equation, and solid mechanics momentum conservation equations. The estimated parameters are within 1% of relative errors compared with the true values in 4 out of 5 test examples and within 2.5% in all examples. The excellent prediction accuracy in our work indicates a promising framework for improving engineering system performance and material designs.

This paper is organized as follows. In Section 2, we first introduce the geometry and governing equations for the classic solid mechanic examples in the present work; we then present an overview of neural network architectures and loss function setup for both soft and hard constraints; we describe the choice of auxiliary functions for hard constraints; at the end, we delineate additional technical details germane to achieving high prediction accuracy in solid mechanics problems. In Section 3, we first demonstrate the effectiveness of various data point sampling strategies; we then compare the performance of soft and hard constraints; subsequently, we present parameter estimation results for both one-dimensional (1D) and 2D examples. Finally, in Sections 4 and 5, we summarize the novelty and benefits of the proposed methods and conclude the paper.

## 2 Methods

In this work, we apply PINNs to estimate material parameters of solid mechanics problems. In addition, we provide details on the neural network architectures, loss functions, BC constraints, and technical considerations specific to solving inverse problems in solid mechanics.

### 2.1 Test examples

We consider five classic examples to cover a spectrum of steady-state and dynamic analyses in 1D and 2D space, representing PDEs of up to fourth order in space and second order in time. The 1D examples were governed by the wave and the Euler-Bernoulli beam equation. The 2D examples were governed by the conservation of momentum, the material constitutive model, and the kinematic relation. The governing equations for each test example are detailed in Appendix A.

**Fig. 1:**
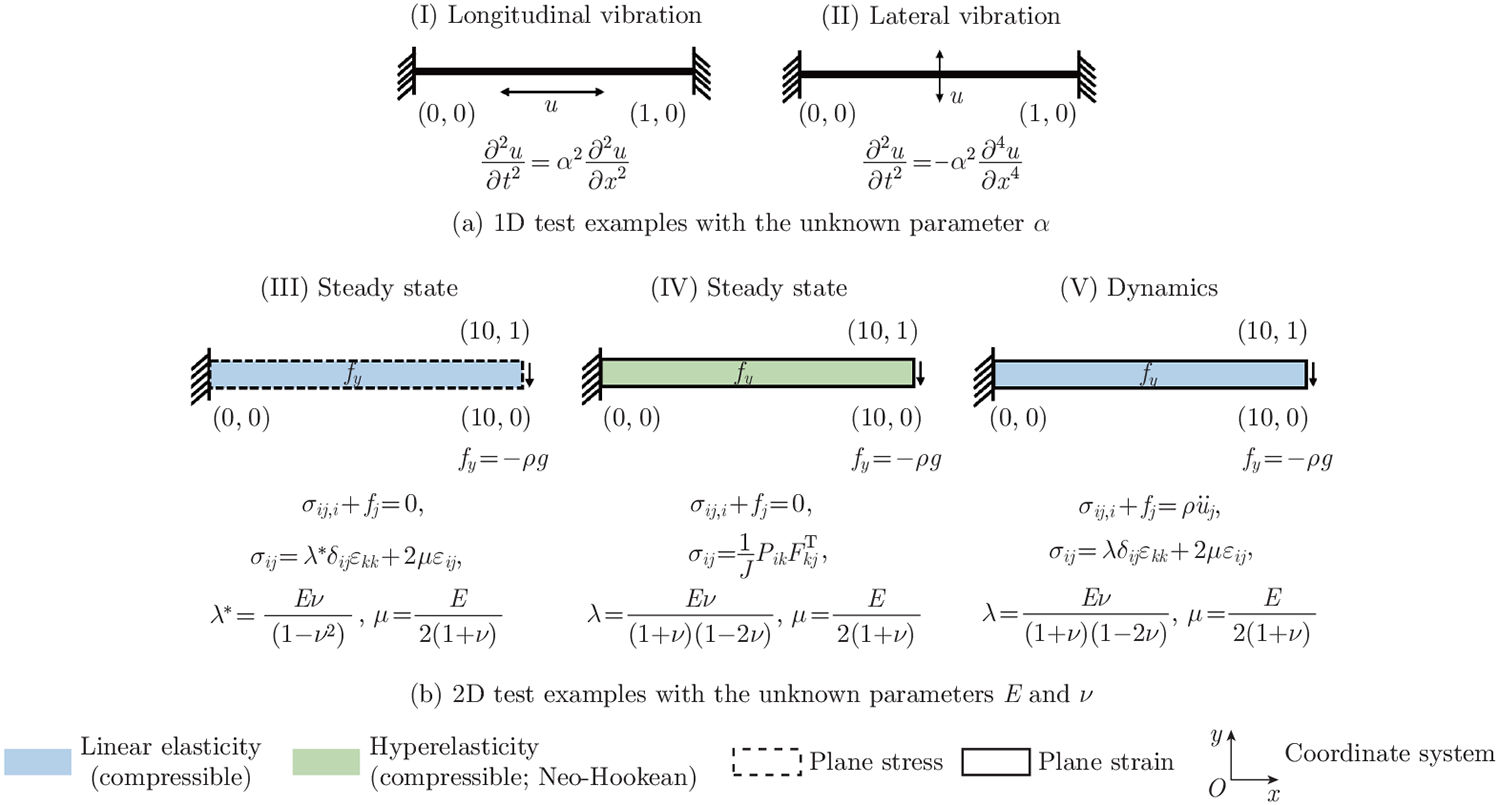
Five classic solid mechanics examples. The geometry of the examples is presented in the undeformed configuration. In addition, we provide the material model, stress configuration, and loading condition for each problem. The aim is to identify the unknown parameter α in the 1D test examples and the unknown parameters E and ν in the 2D test examples. A detailed description of the governing equations is presented in Appendix A

In these inverse problems, we identify the material parameters by utilizing the underlying governing equations and deformation/stress data obtained from either analytical solution or FEA. We provide FEA verification results in Appendix B. To obtain reference data for training, in the 1D examples, we consider a beam that is fixed on both ends. We compute the deformation training data from the analytical solution by applying longitudinal/lateral initial deformation to the beam. In the 2D examples, we consider a cantilever beam that is fixed on the left end. We apply a body force to the structure and use FEA to compute the displacements and stresses on the beam. The details of the model geometry, material model (incompressible linear elastic or compressible hyperelastic material), stress configuration (plane stress or plane strain), and loading condition (applied deformation or body force) are specified in Fig. 1.

In the following, we present the governing equations for a 2D elastodynamic analysis. The momentum equation is expressed as

$$\sigma_{ij,j}+f_{i}=\rho \partial_{tt}u_{i},$$

where ρ is the material density, f is the externally applied force, and the subscript comma denotes partial derivatives. The isotropic linear elastic material constitutive model with plane-strain is

$$\sigma_{ij}=\frac{E\nu }{\left(1+\nu )(1-2\nu \right)}\delta_{ij}\epsilon_{kk}+\frac{E}{\left(1+\nu \right)}\epsilon_{ij}.$$

Herein, E and ν are Young’s modulus and Poisson’s ratio to be estimated using PINNs, respectively, $\delta_{ij}$ is the Kronecker delta, and $\epsilon_{ij}$ is the infinitesimal strain tensor. Lastly, the kinematic relation is written as

$$\epsilon_{ij}=\frac{1}{2}(u_{i,j}+u_{j,i}).$$

### 2.2 PINNs for solid mechanics

Here, we provide an overview of the PINN formulation for solving inverse problems in solid mechanics. The inverse PINN framework was set up using the DeepXDE library^[25]^, and the code is publicly available from the GitHub repository (see https://github.com/lu-group/pinn-material-identification).

#### 2.2.1 Neural network architectures

Let ${𝒩}^{L}(x)\;:\;R^{\text{dim}(x)}\to R^{\text{dim}(y)}$ be an L-layer neural network that maps input features x to output variables y with ${𝒩}^{l}$ neurons in the l-layer. The connectivity between the layers l and l−1 is governed by ${𝒩}^{l}(x)=\phi (W^{l}{𝒩}^{l-1}(x)+b^{l})$, where ϕ is a nonlinear activation function, $W^{l}$ is a weight matrix, and $b^{l}$ is a bias vector. We use hyperbolic tangent, tanh, as the activation function in the present work. Given that the activation function is applied element-wise to each neuron, the recursive fully-connected neural network (FNN) is defined as

$$\text{input layer}\;:\;{𝒩}^{0}(x)=x\in R^{\text{dim}(x)},\;\text{hidden layer}\;l\;:\;{𝒩}^{l}(x)=\text{tanh}\;(W^{l}{𝒩}^{l-1}(x)+b^{l})\in R^{{𝒩}^{l}}\;\text{for}\;1\leq l\leq L-1,\;\text{output layer}\;:\;{𝒩}^{L}(x)=W^{L}{𝒩}^{L-1}(x)+b^{L}\in R^{\text{dim}(y)}.$$

**Fig. 2:**
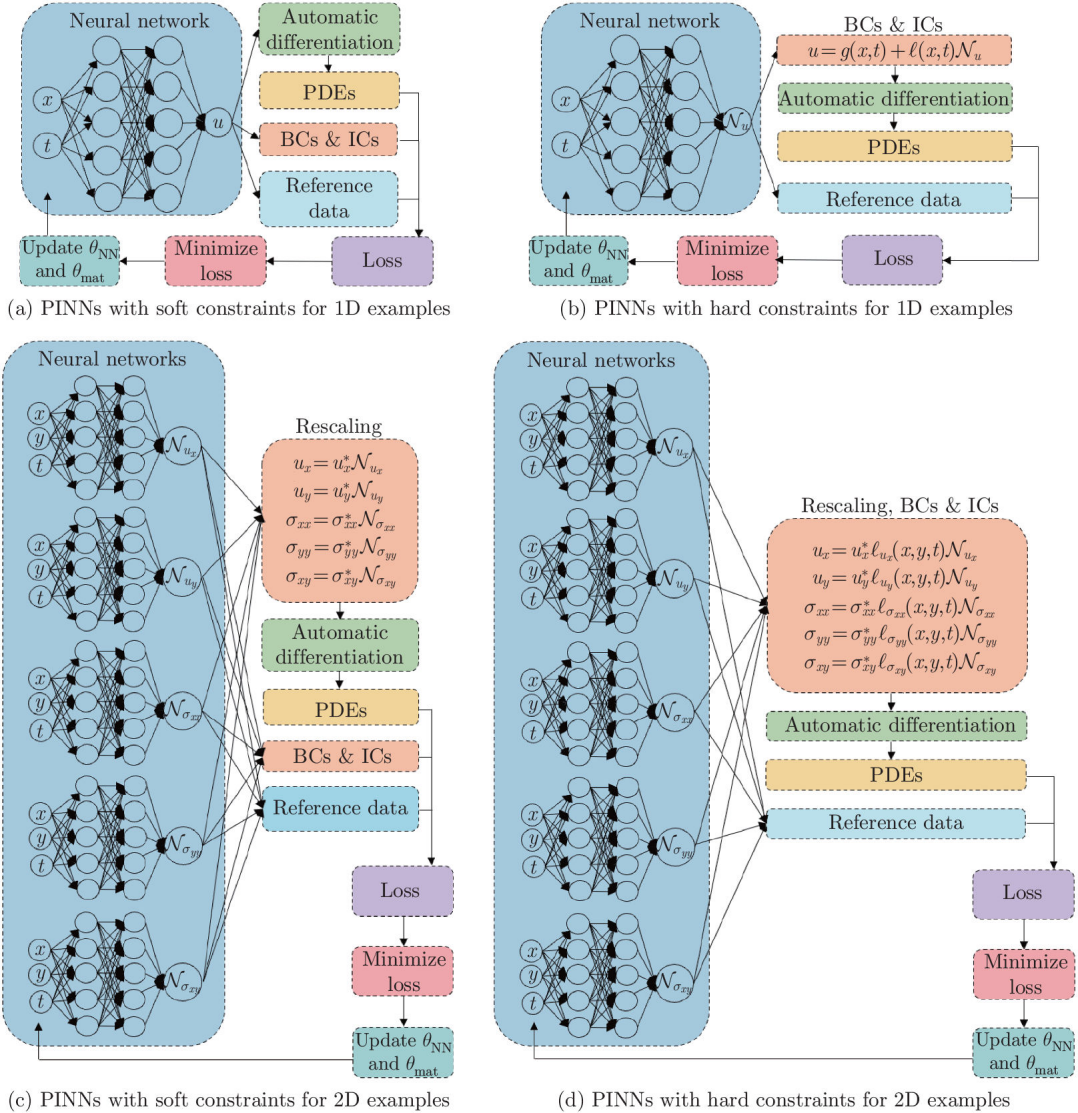
PINN architectures for nonlinear solid mechanics systems. We use one FNN for the 1D examples ((a) and (b)) and five independent FNNs for the 2D examples ((c) and (d)). We consider soft and hard constraints for both 1D and 2D examples

The PINN architectures are presented in Fig. 2. In the 1D examples (see Figs. 2(a) and 2(b)), (x, t) are network input features corresponding to x-Cartesian coordinate and time, and u is the predicted displacement. In the 2D examples (see Figs. 2(c) and 2(d)), (x, y, t) are network input features corresponding to x- and y-Cartesian coordinates and time. Note that the steady-state cases only have two input features, namely x- and y-Cartesian coordinates. (${𝒩}_{u_{x}}$, ${𝒩}_{u_{y}}$, ${𝒩}_{\sigma_{xx}}$, ${𝒩}_{\sigma_{yy}}$, ${𝒩}_{\sigma_{xy}}$) are network outputs. ($u_{x}$, $u_{y}$, $\sigma_{xx}$, $\sigma_{yy}$, $\sigma_{xy}$) are the predicted displacements and stresses. In all four architectures, $\theta_{\text{NN}}$ represents the network parameters ($W^{l}$ and $b^{l}$), and $\theta_{\text{mat}}$ represents the unknown material variables (α for 1D examples, and E and ν for 2D examples).

For complex problems, the network needs to be sufficiently large in order to capture the cross-dependence between variables in the governing equations. Aside from creating a single large network, an alternative approach is establishing a separate, independent network for each output variable. In the present work, we model the test examples using multiple independent networks, given their effectiveness over the single network approach^[27]^. In cases with only one output variable, the network architecture reduces to a standard single FNN. In other words, one FNN is used in the time-dependent 1D vibration test cases, and five independent parallel FNNs are used in the 2D cantilever beam test cases.

We briefly summarize the training procedure of both architectures (see Fig. 2). We first initialize network parameters ($\theta_{\text{NN}}$) to obtain an initial estimation of displacements and/or stresses. Given the initial estimation, we subsequently formulate a loss function to minimize the PDE residuals, as well as the errors between the ground truth and the approximation in the boundary, the initial domain, and reference data points (also known as observational data points). The network variables ($\theta_{\text{NN}}$ and $\theta_{\text{mat}}$) are then updated iteratively until the total loss converges, and the estimated material constants reach a plateau.

#### 2.2.2 Loss functions

In the training process, we optimize the network parameters $\theta_{\text{NN}}$ (i.e., $W^{l}$ and $b^{l}$) and the unknown material parameters $\theta_{\text{mat}}$ (i.e., E and ν), which are expressed as

$$\theta_{\text{NN}}^{*},\theta_{\text{mat}}^{*}=\underset{\theta_{\text{NN}},\theta_{\text{mat}}}{\text{arg min}}ℒ(\theta_{\text{NN}},\theta_{\text{mat}}),$$

where $ℒ(\theta_{\text{NN}},\theta_{\text{mat}})$ is the loss function that measures the total error between network outputs concerning the model’s ICs, BCs, PDE evaluations (conservation of momentum and constitutive material laws) on the collocation points, and the reference data. The total loss function is defined as

$$ℒ(\theta_{\text{NN}},\theta_{\text{mat}})=w_{\text{BCs}}{ℒ}_{\text{BCs}}+w_{\text{ICs}}{ℒ}_{\text{ICs}}+w_{\text{Data}}{ℒ}_{\text{Data}}+w_{\text{PDEs}}{ℒ}_{\text{PDEs}},$$

where w is the weight associated with its corresponding loss term ℒ. The loss terms ${ℒ}_{\text{BCs}}$, ${ℒ}_{\text{ICs}}$, and ${ℒ}_{\text{Data}}$ compute the mean squared errors of predicted results on the collocation points for the BCs, ICs, and observation data, respectively. Further, ${ℒ}_{\text{PDEs}}$ computes the mean squared error of the PDE residuals over the spatial-temporal domain.

In the time-dependent longitudinal and lateral vibration examples, the PDE loss constitutes the residuals in the wave equation and Euler-Bernoulli beam equation. In the 2D examples, the PDE loss comprises the residuals in the constitutive relations and momentum conservation laws. Detailed formulations of the material models and the governing PDEs are presented in Appendix A. The loss function formulation varies slightly depending on the nature of the example problems (i.e., steady state or dynamics). For a 2D linear elastic dynamic example, ${ℒ}_{\text{PDEs}}$ is formulated as

$${ℒ}_{\text{PDEs}}={\left\|\frac{\partial \sigma_{xx}}{\partial x}+\frac{\partial \sigma_{xy}}{\partial y}+f_{x}-\rho \frac{\partial^{2}u_{x}}{\partial t^{2}}\right\|}_{2}^{2}\;\;+\|\frac{\partial \sigma_{xy}}{\partial y}+\frac{\partial \sigma_{yy}}{\partial y}+f_{y}-\rho \frac{\partial^{2}u_{y}}{\partial t^{2}}{\|}_{2}^{2}\;\;+\|\sigma_{xx}-\frac{E_{\text{pred}}}{\left(1+\nu_{\text{pred}})(1-2\nu_{\text{pred}}\right)}((1-\nu_{\text{pred}})\epsilon_{xx}+\nu_{\text{pred}}\epsilon_{yy}){\|}_{2}^{2}\;\;+\|\sigma_{yy}-\frac{E_{\text{pred}}}{\left(1+\nu_{\text{pred}})(1-2\nu_{\text{pred}}\right)}((1-\nu_{\text{pred}})\epsilon_{yy}+\nu_{\text{pred}}\epsilon_{xx}){\|}_{2}^{2}\;\;+\|\sigma_{xy}-\frac{E_{\text{pred}}}{\left(1+\nu_{\text{pred}}\right)}\epsilon_{xy}{\|}_{2}^{2}.$$

In order to compute the PDE loss ${ℒ}_{\text{PDEs}}$, we require higher-order derivatives of the network output (i.e., the displacements) both in time and space. The partial derivatives in the governing equations are approximated using a technique called automatic differentiation. This method evaluates the derivatives by applying the chain rule during back-propagation^[25,50]^. This approach has been shown to be more efficient than conventional numerical methods for estimating derivatives (i.e., finite difference, symbolic differentiation, etc.)^[51-52]^.

In some cases, we consider hard constraint ICs and BCs. Meaning, we impose the ICs and BCs on the network outputs before evaluating the governing equations^[47]^. More information on hard constraints will be discussed in Subsection 2.3. As such, the loss function is simplified into two loss terms,

$$ℒ(\theta_{\text{NN}},\theta_{\text{mat}})=w_{\text{Data}}{ℒ}_{\text{Data}}+w_{\text{PDEs}}{ℒ}_{\text{PDEs}}.$$

The values of the weights for each example are listed in Appendix A.

### 2.3 Hard constraint ICs and BCs

In PINNs, ICs and BCs are usually weakly imposed as soft constraints due to simplicity. However, soft constraints do not guarantee the approximate solution to satisfy the ICs and BCs, which can affect the accuracy of the parameter prediction. Hence, we also impose ICs and Dirichlet-type BCs via hard constraints^[47]^ to ensure the PDEs satisfy the ICs and Dirichlet-type BCs exactly.

Let us consider 𝒩(x) to be the network output, and we aim to satisfy the ICs and Dirichlet-type BCs such that

$${𝒩}^{\prime }(x)=g_{0}(x),\;\;x\in \Gamma_{D}\cup \Omega_{0},$$

where $\Gamma_{D}\subset \partial \Omega$ is a subset of the boundary, and $\Omega_{0}$ is the initial domain. The hard constraint is achieved by using two auxiliary functions g(x) and ℓ(x) (see Figs. 2(b) and 2(d)) such that

$${𝒩}^{\prime }(x)=g(x)+\ell (x)𝒩(x).$$

Here, g(x) is a function that satisfies the required ICs and BCs (which could be either zero or non-zero). Further, ℓ(x) is a function that satisfies the following conditions:

{ℓ(x)=0,x∈ΓD∪Ω0,ℓ(x)>0,x∈Ω∖(ΓD∪Ω0).}

Interested readers can refer to Ref. [47] for more information on defining suitable auxiliary functions.

In this study, we compare the performance of soft constraints and hard constraints. The choice of ℓ(x) is not unique, and based on our experience, it influences the prediction accuracy. For instance, in the 2D examples, we consider two continuous functions and a discontinuous function for ℓ(x) and compare their performance. For the continuous functions, we choose g(x)=0 and ℓ(x)=x, and g(x)=0 and $\ell (x)=\frac{2e^{x}}{e^{x}+1}-1$. For the discontinuous function, we choose g(x)=0 and $\ell (x)=1_{\left\{x>0\right\}}$. All the three hard constraint options outperform the soft constraint. Among the hard constraints, the discontinuous and sigmoid auxiliary functions yield the best results; further research is required to better understand the effect of the auxiliary function characteristics on prediction accuracy.

### 2.4 Additional technical details

In many solid mechanics problems, the displacements and stresses are often several orders of magnitude different from O(1). This wide spread of magnitude orders can present a challenge for the network training process. In the 2D examples with g(x)=0, to achieve an accurate solution, we scale the network outputs (displacement and stresses) by their corresponding maximum absolute values obtained from the observation data, via an output transformation function. This step not only ensures that the magnitude of the network outputs is of O(1), but also circumvents the need to non-dimensionalize the PDEs, as the output variables are mapped to their physical quantities before computing the PDE loss. After transforming the network outputs, we apply hard constraints to the variables,

$$u_{i}=u_{i}^{*}\ell (x){𝒩}_{u_{i}},\;\sigma_{ij}=\sigma_{ij}^{*}\ell (x){𝒩}_{\sigma_{ij}}.$$

Here, ($u_{x}^{*}$, $u_{y}^{*}$, $\sigma_{xx}^{*}$, $\sigma_{yy}^{*}$, $\sigma_{xy}^{*}$) (see Figs. 2(c) and 2(d)) are the maximum absolute displacements and stresses from observational data used for variable scaling.

When approximating unknown variables, the estimated values can converge to a local minimum or trivial solution that satisfies the governing equations but are unrealistic in the physical system. As such, we use a tanh function to constrain the approximation to a realistic range of values. This modification helps guide the network toward meaningful estimations. In particular, in the 2D dynamic example, we set

$$E_{\text{pred}}=5\times 10^{6}\times (\tan h\;{\hat{E}}_{\text{pred}}+1),\;\;\nu_{\text{pred}}=\frac{1}{4}(\tan h\;{\hat{\nu }}_{\text{pred}}+1),$$

where ${\hat{\left(\cdot \right)}}_{\text{pred}}$ is an auxiliary variable, and $(\cdot {)}_{\text{pred}}$ is a scaled variable for use in the PDE calculation. This conversion ensures that all network-predicted auxiliary variables are of a similar scale, which helps improve solution convergence.

## 3 Results

Before we apply PINNs to the examples, we first examine suitable observation point sampling and boundary constraint methods, detailed in Subsections 3.1 and 3.2. After we identify an optimal observation point sampling strategy and boundary constraint method, we apply the optimal composition to estimate the unknown parameters in each test case. The results are reported in Subsections 3.3 and 3.4.

### 3.1 Observation point sampling

#### 3.1.1 1D vibration examples

**Fig. 3:**
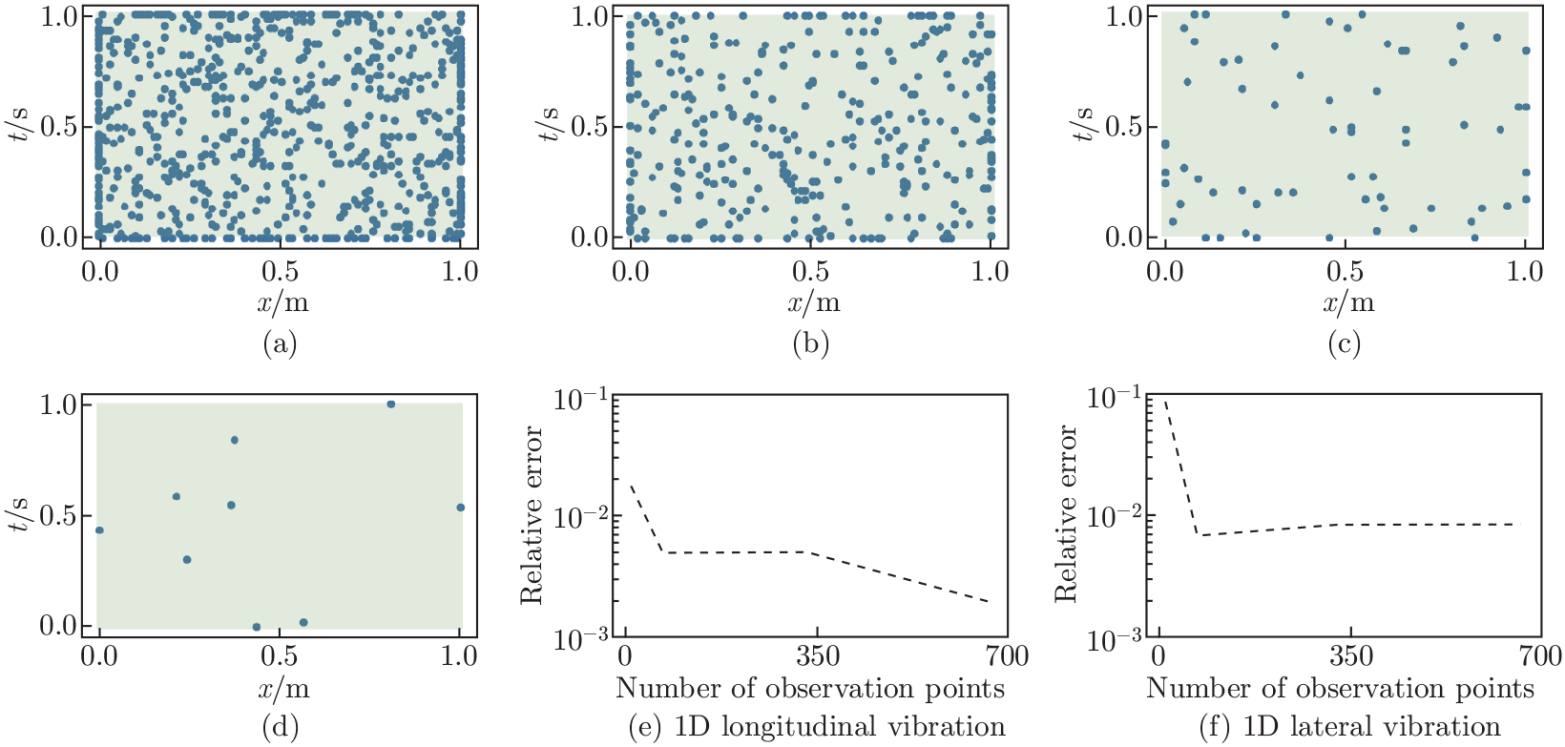
Observation point sampling convergence study. We consider four sets of observation points to investigate the effects of sampling point density on parameter prediction accuracy: (a) 660 observation points; (b) 330 observation points; (c) 66 observation points; (d) 9 observation points. In both (e) and (f), the error reduces with increasing the number of observation points

In the two 1D vibration examples, we test the accuracy of the estimated $\alpha_{\text{pred}}$ by computing the relative errors for four sets of sampling points with varying densities. These sampling points are shown in Figs. 3(a)-3(d). We run the longitudinal and lateral vibration examples for each sampling point test, using 500 thousand and 1 million iterations, respectively. At the final iteration, we record the relative errors and plot them versus the number of observation points in Figs. 3(e) and 3(f). To sample the PDE collocation points, we use random sampling in both examples. Specifically, in the longitudinal vibration example, we use $N_{d}^{\text{PDEs}}=20$ (spatial-temporal domain points), $N_{b}^{\text{PDEs}}=10$ (boundary points), and $N_{i}^{\text{PDEs}}=10$ (initial temporal points). Meanwhile, in the lateral vibration example, we use $N_{d}^{\text{PDEs}}=100$ (spatial-temporal points), $N_{b}^{\text{PDEs}}=50$ (boundary points), and $N_{i}^{\text{PDEs}}=50$ (initial temporal points).

In Figs. 3(e) and 3(f), we observe that the increase in the observation points leads to a reduction in the relative error by almost an order of magnitude, with the most significant drop occurring from 9 to 66 sample points in both examples; the relative errors are relatively steady afterward. In the longitudinal vibration example, the relative error reduces from 1.8% to 0.2% as we increase the sampling data from 9 to 660 observation points, while in the lateral vibration example, it decreases from 8.68% to 0.85%. It is worth noting that the relative errors in the lateral vibration example are consistently higher than those in the longitudinal example. This difference in errors can be attributed to the Euler-Bernoulli beam equation having a higher-order derivative (a PDE of fourth order in space and second order in time ).

#### 3.1.2 2D steady-state examples

**Fig. 4:**
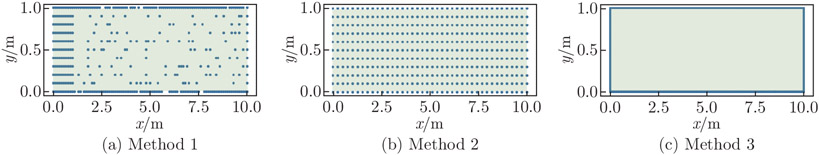
Observation point sampling for the 2D steady-state examples. We investigate the effects of three observation point sampling strategies on parameter prediction accuracy for the 2D steady-state examples. A total of 440 observation points are sampled in each of the three methods. (a) In Method 1, we concentrate sampling points near the fixed end of the beam. (b) In Method 2, we place sampling points uniformly over the spatial domain. (c) In Method 3, we sample the boundary points only

In our experimentation, we discover that the observation point distribution plays a significant role in estimating the unknown parameters in the 2D examples. As such, we perform a comparative study on the observation point sampling strategies to evaluate their influence on the unknown parameter prediction for the 2D steady-state problems to identify an efficient sampling approach. We consider three methods (see Fig. 4). In Method 1, we sample 121 points near the fixed boundary bounded by x∈[0,1]m and y∈[0,1]m, and 129 observation points in the rest of the interior (x, y) region. In addition, we sample a total of 190 points on the top, bottom, and right boundaries. In Method 2, we uniformly distribute 440 observation points in the spatial domain. Finally, in Method 3, we sample a total of 440 boundary points on the top, bottom, left, and right edges. Across all the three methods, we use PDE collocation point sampling with spatial domain points $N_{d}^{\text{PDEs}}=100$ and boundary points $N_{b}^{\text{PDEs}}=50$.

**Fig. 5:**
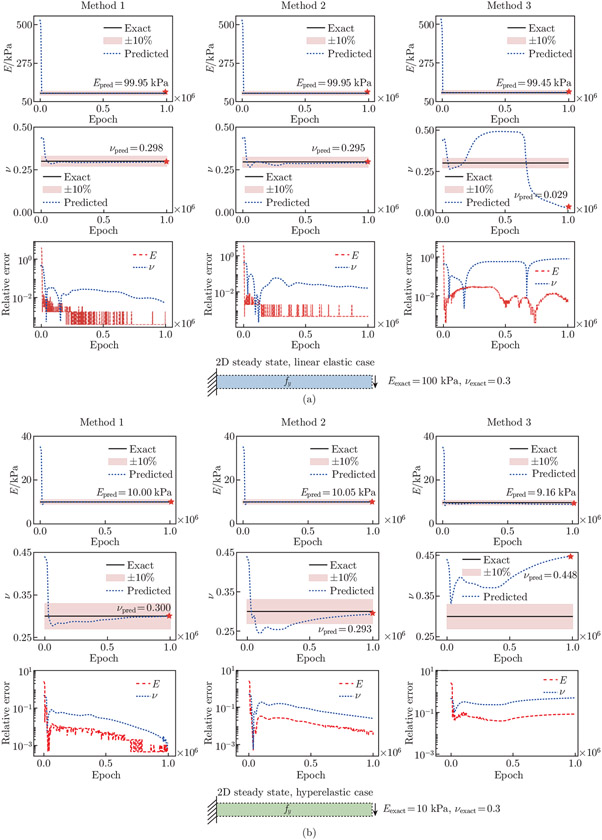
Three observation point sampling strategies for the 2D linear elastic and hyperelastic steady-state examples. The convergence behavior and the relative errors of $E_{\text{pred}}$ and $\nu_{\text{pred}}$ are shown. (a) In the linear elastic case, the relative errors of $E_{\text{pred}}$ and $\nu_{\text{pred}}$ are 0.047% and 0.539% for Method 1, 0.047% and 1.745% for Method 2, and 0.546% and 90.264% for Method 3, respectively. (b) In the hyperelastic case, the relative errors of $E_{\text{pred}}$ and $\nu_{\text{pred}}$ are 0.046% and 0.059% for Method 1, 0.497% and 2.392% for Method 2, and 8.402% and 49.43% for Method 3, respectively

The estimated unknown parameters, $E_{\text{pred}}$ and $\nu_{\text{pred}}$, are shown in Fig. 5. Different observation point sampling methods do not significantly influence the convergence of $E_{\text{pred}}$. We observe that $E_{\text{pred}}$ quickly converges to an exact value in all the three sampling methods for linear elastic and Neo-Hookean test examples. On the other hand, the distribution of observation points has a significant effect on $\nu_{\text{pred}}$; $\nu_{\text{pred}}$ fails to converge in Method 2 (uniformly distributed observation points) and Method 3 (observation points on the boundary only). This inconsistency is due to the fact that, by Saint Venant’s principle, Poisson’s effect is insensitive in the regions far from the boundaries. In the cantilever beam stress profiles, we observe concentrated stresses near the fixed end, and the stresses rapidly decay to close to zero in the free end. As such, observation points away from the fixed end may not provide enough information for the network to recover the true value of ν, especially in the cases where the magnitude order of ν is substantially smaller than that of E.

#### 3.1.3 2D dynamic example

In the 2D dynamic example, we perform a comparative study to examine the accuracy of the predicted parameters with various amounts of temporal reference data. In Method 1, we extract reference data from 11 time frames with t=[0,0.1,0.2,⋯,0.8,0.9,1]s in the FEA displacement and stress fields. In Method 2, we extract reference data from 6 time frames with t=[0,0.1,0.2,0.3,0.4,0.5]s. Finally, in Method 3, we extract reference data from 3 time frames with t=[0,0.1,0.2]s. For each method, we run the inverse analysis with 5 independent networks. Each network has 3 hidden layers, with 20 neurons per layer.

**Fig. 6:**
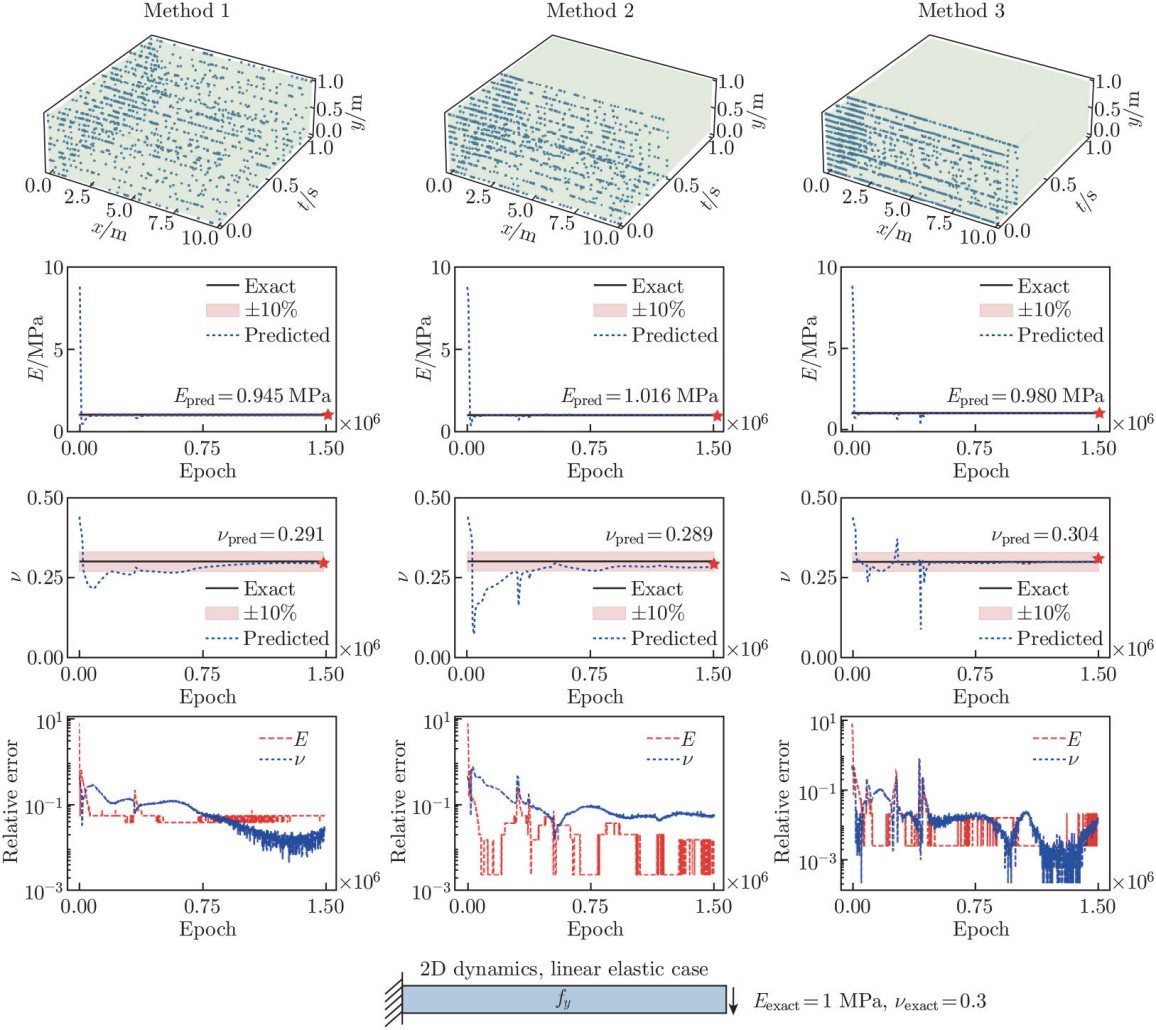
Time series sampling. We consider three time series sampling strategies. The convergence behavior and the relative errors of $E_{\text{pred}}$ and $\nu_{\text{pred}}$ are shown. In Method 1, we extract 11 time frames from the FEA displacement and stress fields to use as the reference data. The relative errors for $E_{\text{pred}}$ and $\nu_{\text{pred}}$ are 5.510% and 2.959%, respectively. In Method 2, we extract 6 time frames. The relative errors for $E_{\text{pred}}$ and $\nu_{\text{pred}}$ are 1.561% and 5.321%, respectively. In Method 3, we extract 3 time frames. The relative errors for $E_{\text{pred}}$ and $\nu_{\text{pred}}$ are 2.031% and 1.378%, respectively. Our networks are able to achieve satisfactory $E_{\text{pred}}$ and $\nu_{\text{pred}}$ using the reference data from as little as 3 time frames

The estimated unknown parameters, $E_{\text{pred}}$ and $\nu_{\text{pred}}$, are shown in Fig. 6. As demonstrated, we achieve satisfactory $E_{\text{pred}}$ and $\nu_{\text{pred}}$ using reference data from as little as 3 time frames (the first time frame is the ICs). Similar to Subsection 3.1.2, the estimation of $E_{\text{pred}}$ is not affected by the time series sampling method; reducing the volume of reference data in the temporal domain does not have an adverse effect on $E_{\text{pred}}$. On the contrary, our results demonstrate improved accuracy in $\nu_{\text{pred}}$ when fewer reference data are used. One reason could be that, as the volume of reference data increases, one would need sufficiently large networks to capture the interdependency between variables. However, given that the unknown parameters in our application are time-independent, all time frames in the training process are unnecessary. As such, it is found that Method 3 is an optimal choice, as it produces the most accurate predictions and is the most computationally efficient among the three methods.

#### 3.1.4 Summary of observation point sampling strategy

**Fig. 7:**
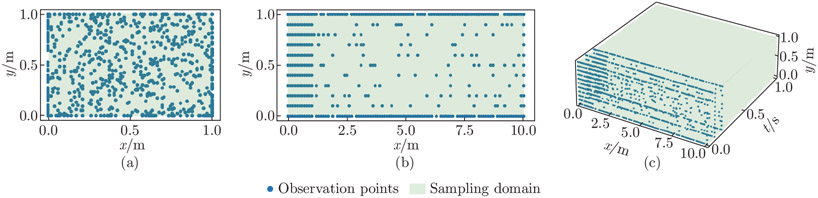
Observation point distribution. (a) We use a total of 660 observation points for the vibration examples. Among those, 160 points are randomly distributed along the boundaries, and 500 points are randomly distributed in the interior region. (b) We use a total of 440 observation points for the 2D steady-state examples. Among those, 121 points are concentrated near the fixed boundary bounded by x≤1m; 129 points are sampled in the region x>1; and 190 points are sampled on the top, bottom, and right boundaries. (c) We follow a similar point distribution strategy as (b) to extract the observation point coordinates at time instances t=[0,0.1,0.2]s; a total of 1 099 observation points are sampled for the dynamic example

The observation point sampling technique used in the present work is summarized in Fig. 7. For the 1D examples, we randomly distribute 160 points along the boundary bounded by x∈[0,1]m and t∈[0,1]s and 500 points in the interior domain. We then compute the analytical solutions given their (x, t) coordinates at the observation points. The analytical solution for longitudinal vibration is $u^{*}=\sin(\pi x)\;\cos(\pi t)$. Meanwhile, the analytical solution for lateral vibration is $u^{*}=\sin(\pi x)\;\cos(\pi^{2}t)$. For the 2D steady-state examples, we sample 121 points near the fixed boundary bounded by x∈[0,1]m and y∈[0,1]m, and randomly distribute 129 observation points in the remaining (x, y) region. In addition, we sample an additional 190 points randomly distributed on the top, bottom, and right boundaries. Lastly, for the 2D dynamic example, we follow a similar sampling strategy as the steady-state examples. Since both E and ν are constant in time and given PINN’s ability to uncover material parameters from incomplete data, it is unnecessary to use observation points from the entirety of t∈[0,1]s. In the present work, the observation points are extracted at time instances t=[0,0.1,0.2]s. The displacements and stresses at the observation points are used as the reference data. The number of sample points is chosen arbitrarily in this work.

### 3.2 Boundary constraint studies

Here, we examine the unknown parameter prediction accuracy using soft and hard constraints. In soft constraints, the BCs are enforced directly during the constrained optimization process by introducing a loss term in the loss function; the governing PDEs are guaranteed to satisfy the BCs. Meanwhile, in the hard constraints, the neural network architecture is modified such that the BCs are explicitly enforced (via an auxiliary function) before computing the PDE loss. This ensures that the BCs are satisfied precisely in the training process. The governing equation for this example constitutes a fourth-order spatial partial derivative and a second-order temporal partial derivative. The higher-order spatial and temporal derivatives in the PDEs amplify noise in the training process, which makes identifying the unknown parameter in this example a challenging task^[53-54]^.

#### 3.2.1 1D lateral vibration example

**Fig. 8:**
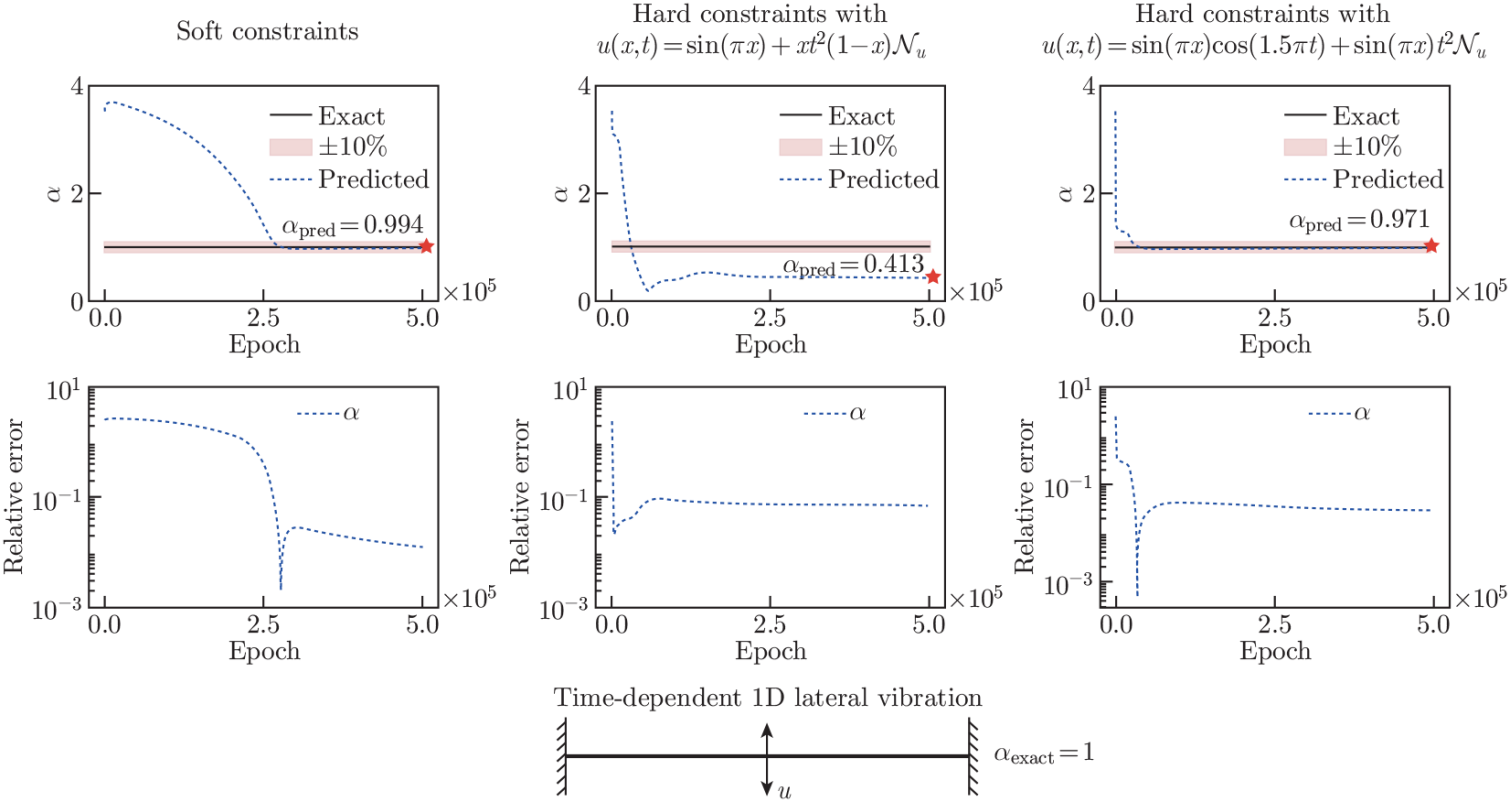
Boundary constraints for the 1D lateral vibration example. We compare the accuracy of $\alpha_{\text{pred}}$ using soft constraints and hard constraints. We consider two hard constraint auxiliary functions. The convergence behavior and the relative errors of $\alpha_{\text{pred}}$ are shown. The results indicate that the choice of auxiliary function significantly influences the accuracy of $\alpha_{\text{pred}}$. It is found that soft constraints produce the most accurate estimation of $\alpha_{\text{pred}}$

In Fig. 8, we compare the accuracy of the parameter $\alpha_{\text{pred}}$ using soft constraints and hard constraints. We test three different hard constraint auxiliary functions in this example; the auxiliary functions are formulated such that they satisfy the BCs stated in Appendix A. Although the literature has shown that hard constraints offer better predictive power for inverse designs^[47]^, our results indicate that hard constraints’ performance depends on the auxiliary function. Interestingly, it is found that soft constraints outperform hard constraints in this particular example. However, with appropriate auxiliary functions, hard constraints can converge to an acceptable solution faster. The relative error for $\alpha_{\text{pred}}$ achieves 0.55% using soft constraints.

#### 3.2.2 2D elastostatic example

**Fig. 9:**
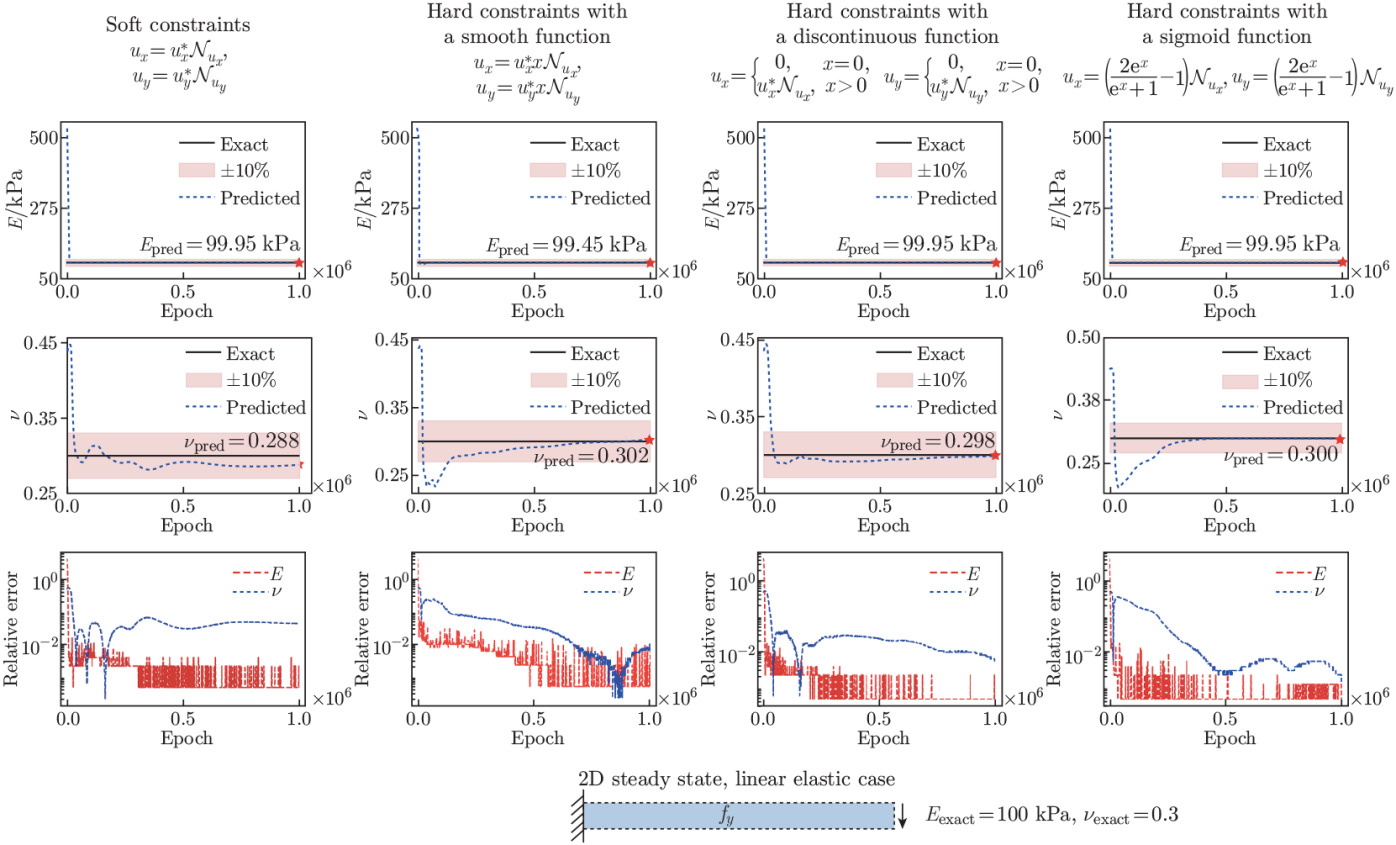
Boundary constraints for the 2D elastostatic example. We examine the accuracy of $E_{\text{pred}}$ and $\nu_{\text{pred}}$ using soft constraints, hard constraints with a smooth function, a discontinuous function, and a sigmoid function. The convergence behavior and the relative errors of $E_{\text{pred}}$ and $\nu_{\text{pred}}$ are shown. In this example, hard constraints with discontinuous and sigmoid functions produce the best estimated $E_{\text{pred}}$ and $\nu_{\text{pred}}$

We compare the e^®^ects of soft and hard constraints on the 2D cantilever beam problem. For the hard constraint auxiliary functions, we select a smooth, discontinuous function, and a sigmoid function to enforce the x- and y-displacement conditions. In Fig. 9, we observe that the convergence characteristic for $\nu_{\text{pred}}$ with a sigmoid auxiliary function is similar to that with a smooth function; both smooth functions require more iterations for $\nu_{\text{pred}}$ to converge within a reasonable range compared with a discontinuous auxiliary function. Hard constraints with discontinuous functions and sigmoid functions provide the best estimations of $E_{\text{pred}}$ and $\nu_{\text{pred}}$. The relative errors for $E_{\text{pred}}$ and $\nu_{\text{pred}}$ with a discontinuous function are 0.047% and 0.539%, respectively; the errors drop slightly to 0.046% and 0.139% with a sigmoid auxiliary function, respectively.

As demonstrated in Subsection 3.2.1, it is found that the soft constraint network architecture is sufficient for accurately identifying the unknown parameter in the time-dependent 1D cases. Meanwhile, PINNs with properly chosen hard constraints^[47]^ cast significant improvement for estimating unknown material constants in the 2D examples. This study again highlights the importance of the auxiliary function selection on the accuracy of unknown parameters for inverse designs.

### 3.3 Parameter estimation result: time-dependent 1D examples

**Fig. 10:**
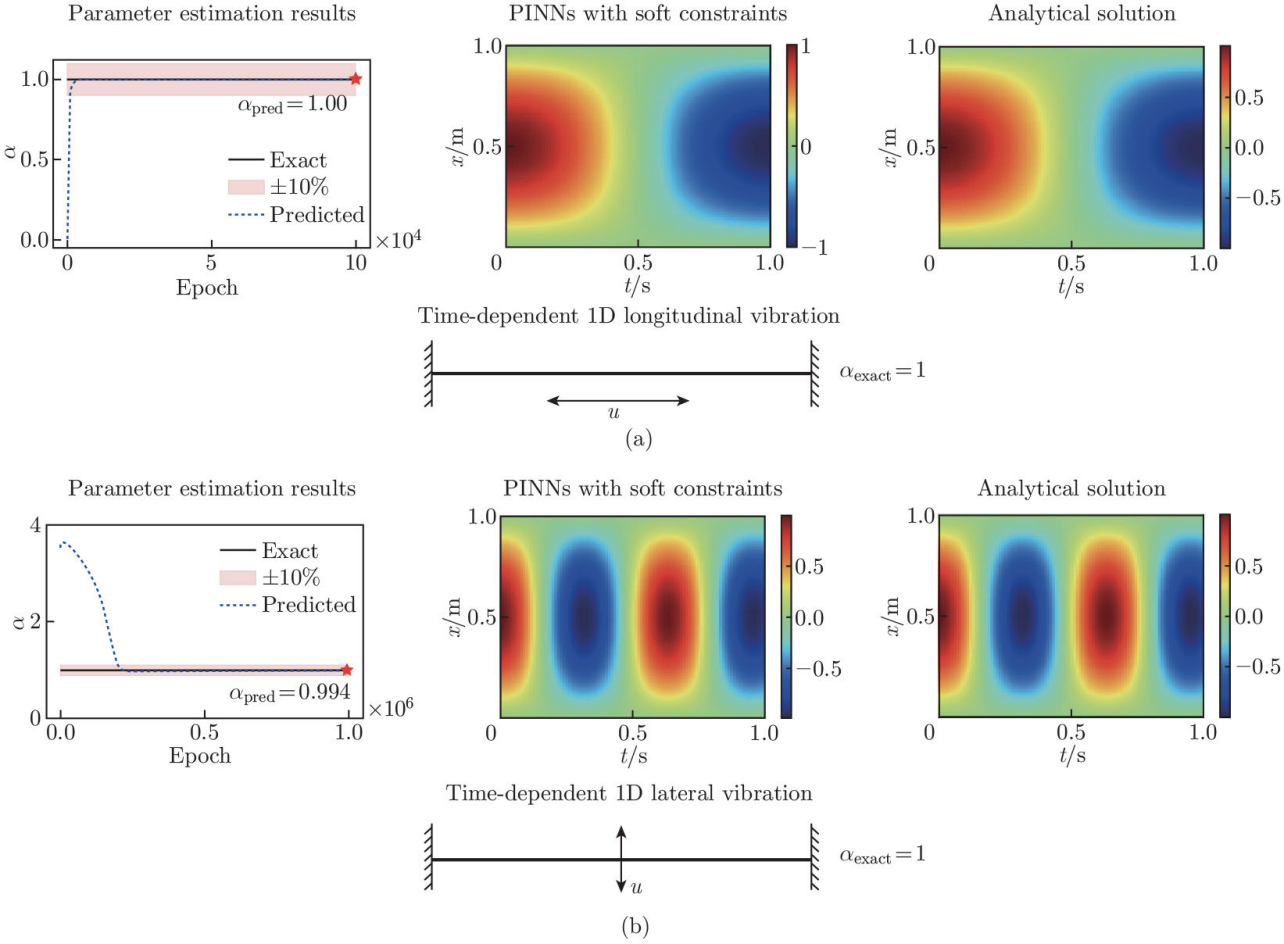
1D vibration parameter estimation results. The best estimated results of the unknown parameter, PINN predictions, and analytical solution of u are provided. (a) The analytical solution for the longitudinal vibration example is $u_{\text{analytical}}=\sin(\pi x)\cos(\pi t)$. PINNs successfully recover $\alpha_{\text{pred}}$ for the time-dependent 1D longitudinal example to the true value; the relative error is 0.00%. (b) The analytical solution for the lateral vibration example is $u_{\text{analytical}}=\sin(\pi x)\cos(\pi^{2}t)$. The relative error of $\alpha_{\text{pred}}$ for the lateral vibration example is 0.55%. The $L_{2}$ relative error of the displacement fields is 2.832%

The network architecture for the 1D examples contains 3 hidden layers, with 50 neurons per layer. We set the learning rate to 10^−3^. In these cases, we aim to estimate the unknown parameter α in the governing equations. In the longitudinal vibration example, we train the network with 100 thousand epochs. We repeat the simulation six times, each time with a varied random seed. The network estimated $\alpha_{\text{pred}}$ converges to the exact value, $\alpha_{\text{exact}}=1$, with a relative error of (0.133 ± 0.082)%. The resulting displacement field recovers the analytical solution. In the lateral vibration example, we train the network with 1 million epochs. Similarly, the simulation is repeated six times with varied random seeds. We use a hyperbolic tangent function to constrain α to ensure that the estimated value is physical. In particular, we constrain $\alpha_{\text{pred}}$ to [0, 4] by setting $\alpha_{\text{pred}}=2(\tan h\;{\hat{\alpha }}_{\text{pred}}+1)$, where ${\hat{\alpha }}_{\text{pred}}$ is an auxiliary variable, and $\alpha_{\text{pred}}$ is a scaled variable for the PDE calculation. The network estimate $\alpha_{\text{pred}}=0.994$, with a (0.731 ± 0.126)% relative error compared with the exact value, $\alpha_{\text{exact}}=1$. The $L_{2}$ relative error of the resulting displacement field is 2.782%. The best parameter estimation results are shown in Fig. 10.

### 3.4 Parameter estimation result: 2D examples

**Fig. 11:**
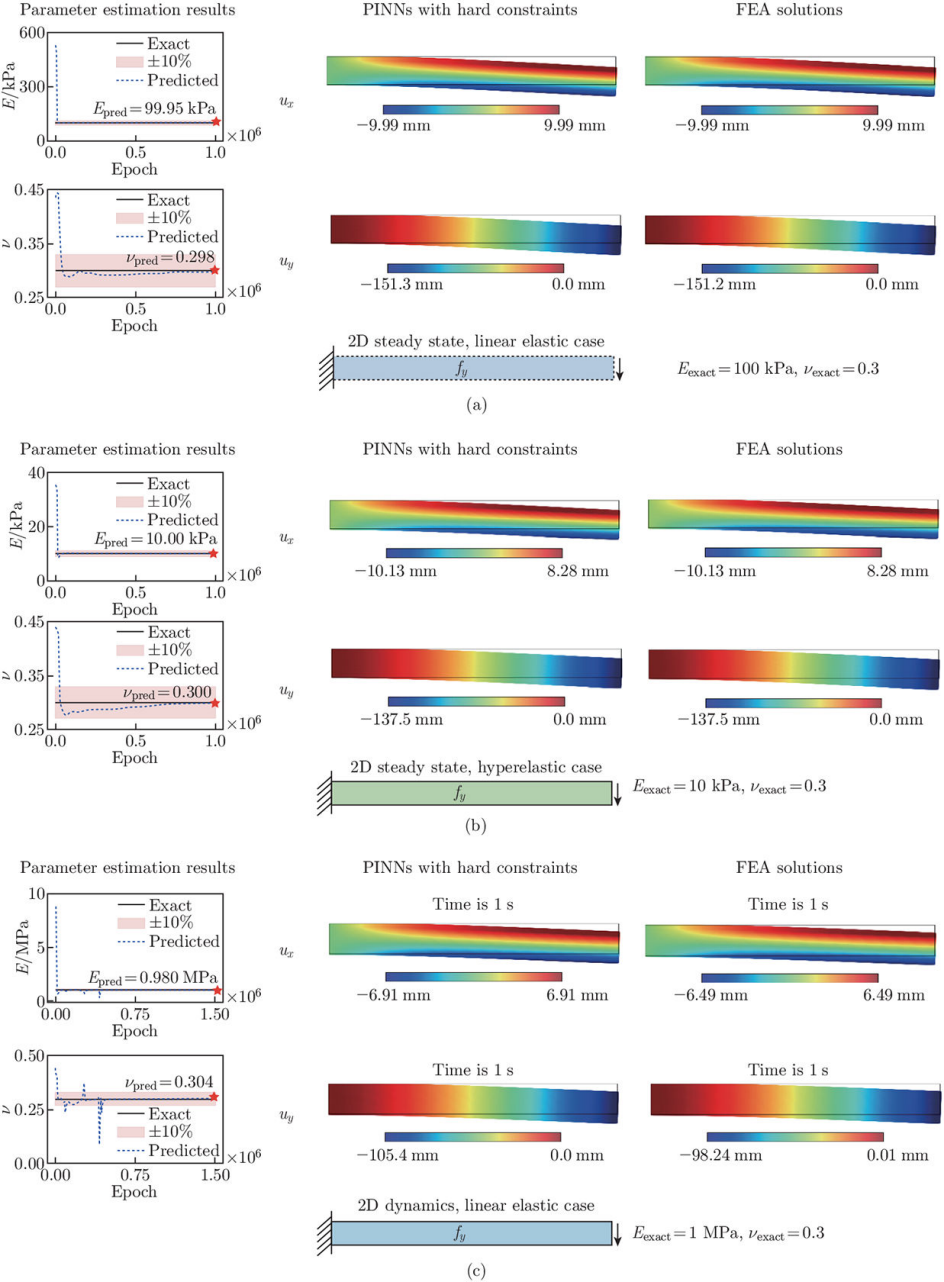
2D cantilever beam parameter estimation and beam displacements. The estimated values of E and ν along with the PINN and FEA approximations of $u_{x}$ and $u_{y}$ are provided. (a) In the 2D linear elastic steady-state example, the relative errors of the estimated $E_{\text{pred}}$ and $\nu_{\text{pred}}$ are 0.047% and 0.539%, respectively. The $L_{2}$ relative errors of the estimated displacement fields $u_{x}$ and $u_{y}$ are 0.049% and 0.048%, respectively. (b) In the 2D hyperelastic steady-state example, the relative errors of $E_{\text{pred}}$ and $\nu_{\text{pred}}$ are 0.046% and 0.059%, respectively. The $L_{2}$ relative errors of the estimated displacement fields $u_{x}$ and $u_{y}$ are both 0.034%. (c) In the 2D linear elastic dynamic example, the relative errors of $E_{\text{pred}}$ and $\nu_{\text{pred}}$ are 2.031% and 1.377%, respectively. The $L_{2}$ relative errors of the estimated displacement fields $u_{x}$ and $u_{y}$ are 3.371% and 3.278%, respectively

**Fig. 12:**
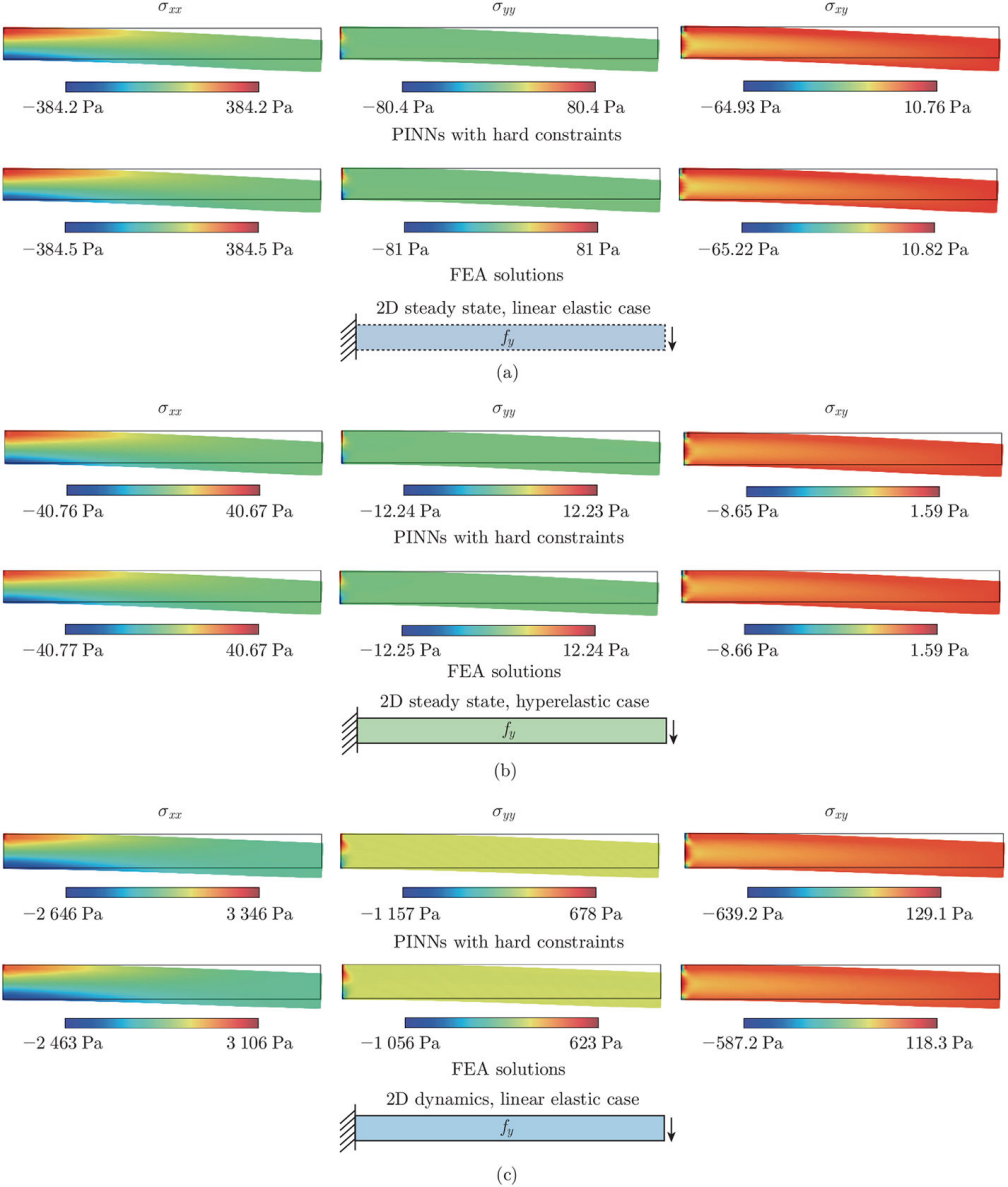
2D cantilever beam stresses. We provide the PINN and FEA approximations of $\sigma_{xx}$, $\sigma_{yy}$, and $\sigma_{xy}$. (a) In the 2D linear elastic steady-state example, the $L_{2}$ relative errors of the estimated stress fields $\sigma_{xx}$, $\sigma_{yy}$, and $\sigma_{xy}$ are 0.018%, 0.553%, and 0.209%, respectively. (b) In the 2D hyperelastic steady-state example, the $L_{2}$ relative errors of the estimated stress fields $\sigma_{xx}$, $\sigma_{yy}$, and $\sigma_{xy}$ are 0.004%, 0.087%, and 0.038%, respectively. (c) In the 2D linear elastic dynamic example, the $L_{2}$ relative errors of the estimated stress fields $\sigma_{xx}$, $\sigma_{yy}$, and $\sigma_{xy}$ at t=1s are 3.084%, 3.388%, and 3.832%, respectively

We use five independent neural networks in the 2D examples. For the steady-state examples, each network has 3 hidden layers, with 15 neurons per layer. For the dynamic example, we use 3 hidden layers per network, with 20 neurons per layer. The performances of various network architectures are presented in Appendix C. We set the learning rate to 10^−3^. In these cases, we aim to estimate the unknown Young’s modulus and Poisson’s ratio, E and ν, in the material constitutive laws. The steady-state models are trained with 1 million epochs. Meanwhile, the dynamic model is trained with 1.5 million epochs. We use double-precision floating point in the 2D examples. For all the three examples, we compute the reference displacements and Cauchy stresses using an open-source finite element software, FEniCS^[55]^, based on the predefined $E_{\text{exact}}$ and $\nu_{\text{exact}}$ values. The approximate $E_{\text{pred}}$ and $\nu_{\text{pred}}$, as well as the displacement and stress fields, are shown in Figs. 11 and 12. The relative errors of $E_{\text{pred}}$ and $\nu_{\text{pred}}$, and the resulting $u_{x}$, $u_{y}$, $\sigma_{xx}$, $\sigma_{yy}$, and $\sigma_{xy}$ are summarized in Table 1.

The differences in the magnitude order between the displacement and stress fields can pose a challenge in the training process and influence solution convergence. In the present work, the displacements range from $O(10^{-1})\;m$ to $O(10^{-3})\;m$, while the stresses range from O(1)Pa to $O(10^{2})\;\text{Pa}$. To improve convergence, we rescale the network output displacements and stresses by their respective maximum absolute values in the reference solution. This helps ensure that the network output variables are all in O(1). Further, the magnitude order disparity between material parameters, E and ν, can also present difficulties in identifying an accurate solution. Similar to the 1D examples (see Subsection 3.3), we use a tanh function to not only ensure that the predicted material constants are in a realistic range but also keep the auxiliary variables predicted by the network in similar magnitude orders.

**Fig. 13:**
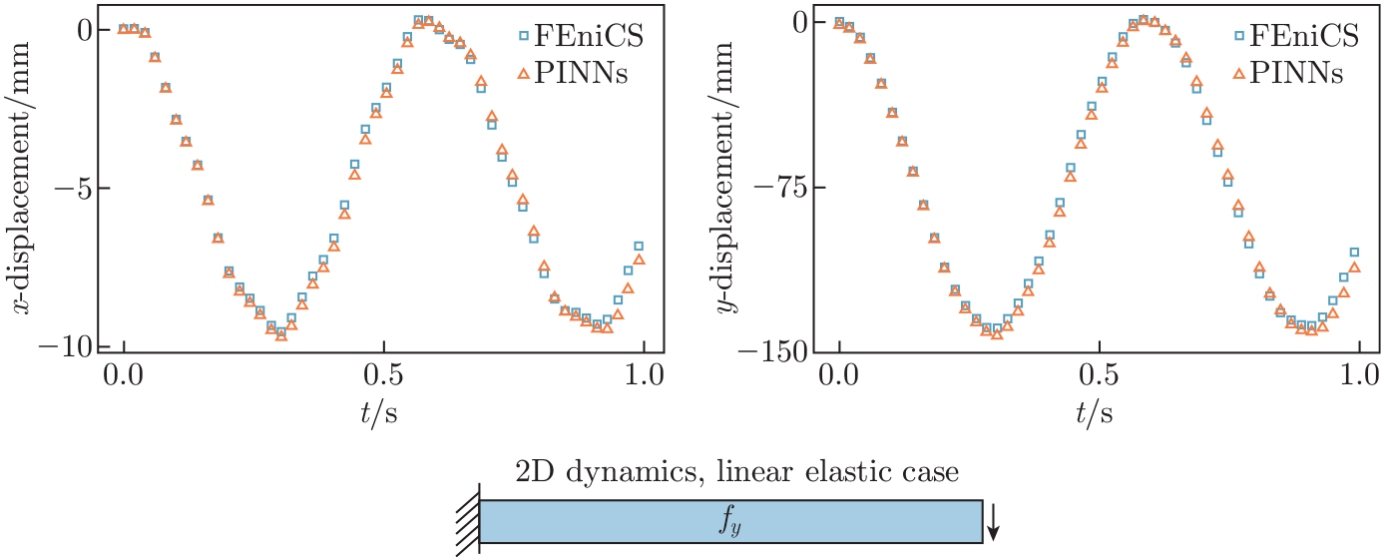
2D cantilever beam tip deflection evolution. We compare the displacement at the cantilever tip against FEniCs for the elastodynamic example. The displacements predicted by PINNs agree with the FEA solution qualitatively, with the PINN predicted displacements exhibiting slightly higher dissipation as time evolves. The $L_{2}$ relative errors of the estimated x- and y-displacements for time t∈[0,1]s are 0.346% and 3.306%, respectively

In the 2D linear elastic steady-state example, we constrain $E_{\text{pred}}$ to [0, 700] kPa and ν to [0, 0.5]. The network estimates $E_{\text{pred}}=99.95\;\text{kPa}$ and $\nu_{\text{pred}}=0.298$, compared with the exact values $E_{\text{exact}}=100\;\text{kPa}$ kPa and $\nu_{\text{exact}}=0.3$. In the 2D hyperelastic steady-state example, we constrain $E_{\text{pred}}$ to [0, 60] kPa and ν to [0,0.5]. The exact values of Young’s modulus and Poisson’s ratio are $E_{\text{exact}}=10\;\text{kPa}$ and $\nu_{\text{exact}}=0.3$, respectively. The predicted values are $E_{\text{pred}}=10.0\;\text{kPa}$ and $\nu_{\text{pred}}=0.300$. In the 2D dynamic example, we constrain $E_{\text{pred}}$ to [0, 10] MPa and ν to [0, 0.5]. The exact values of Young’s modulus and Poisson’s ratio are $E_{\text{exact}}=1$ MPa and $\nu_{\text{exact}}=0.3$, respectively. The predicted values are $E_{\text{pred}}=0.980$ MPa and $\nu_{\text{pred}}=0.304$. The x- and y-displacements at the free end of the cantilever for t∈[0,1]s are presented in Fig. 13. The PINN approximate displacements show excellent alignment with reference data generated from FEniCS. The $L_{2}$ relative errors for the x- and y-tip displacements are 3.462% and 3.306%, respectively.

### 3.5 Transfer learning for inverse problems

**Fig. 14:**
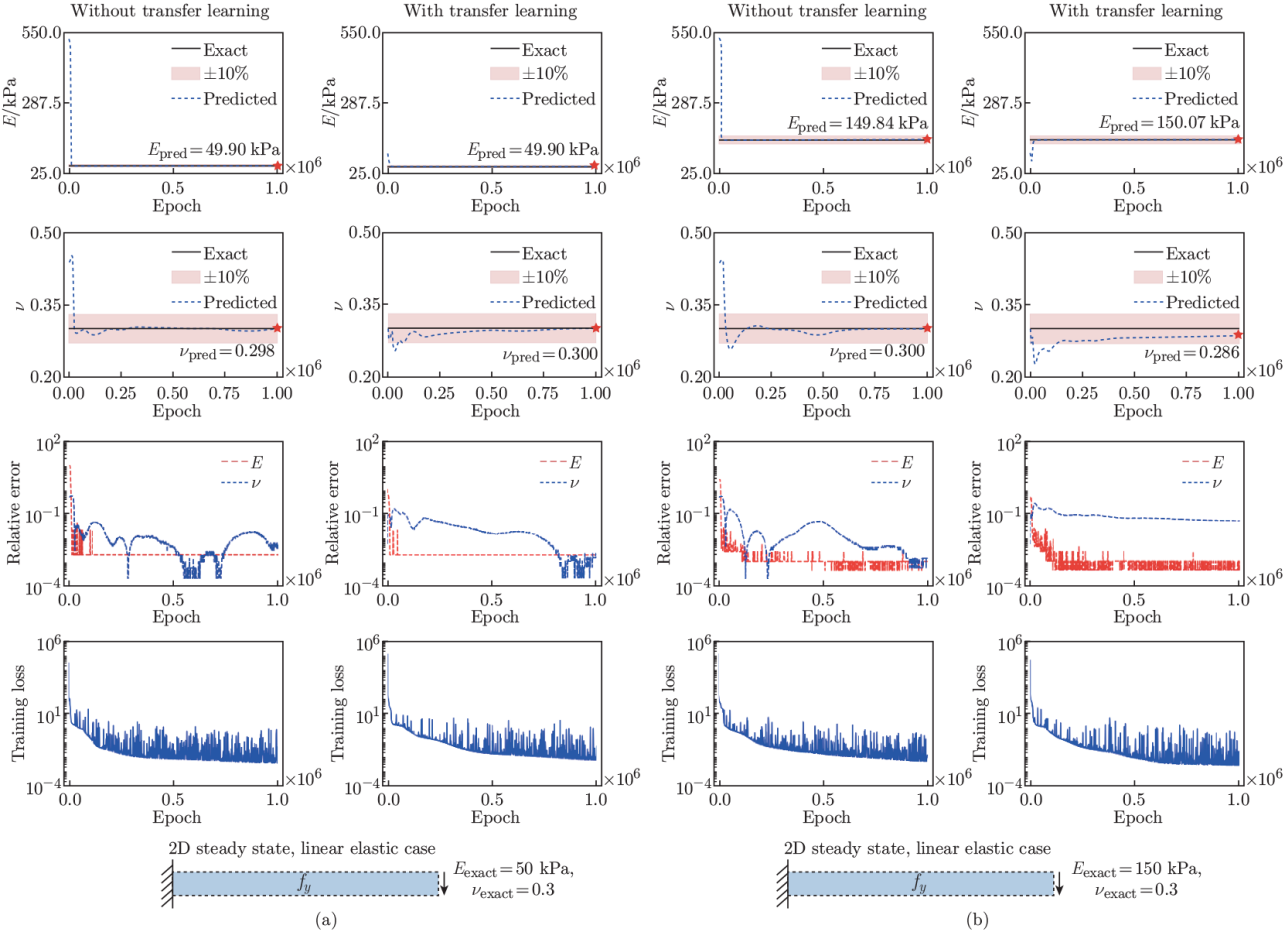
PINN performance on multiple sets of material parameters with and without transfer learning. We apply PINNs to learn the unknown parameters in the linear elastic steady-state problem with exact material parameters of (a) $E_{\text{exact}}=50\;\text{kPa}$ and $\nu_{\text{exact}}=0.3$ and (b) $E_{\text{exact}}=150\;\text{kPa}$ and $\nu_{\text{exact}}=0.3$ to compare the solution accuracy with and without transfer learning. The convergence behavior and relative errors for $E_{\text{pred}}$ and $\nu_{\text{pred}}$, as well as the training loss of the models are provided. While both methods converge to similar $E_{\text{pred}}$ and $\nu_{\text{pred}}$ in (a), it appears that starting training de novo yields better estimate of $\nu_{\text{pred}}$, compared with that with transfer learning in (b)

We have thus far demonstrated the high predictive power of our methods for learning one set of material parameters. To demonstrate the applicability and generality of PINNs to diverse problems, we evaluate the solution accuracy on two additional sets of material parameters for the linear elastic steady-state example (see Fig. 14). Further, we perform each of the analyses with transfer learning to determine its effects on solution accuracy. In transfer learning, we use the network trained with $E_{\text{exact}}=100$ kPa and $\nu_{\text{exact}}=0.3$ to initialize the network in the two cases.

As shown in Fig. 14, our methods are able to successfully uncover a diverse range of material parameters. In the example with $E_{\text{exact}}=50$ kPa and $\nu_{\text{exact}}=0.3$, the relative errors of $E_{\text{pred}}$ and $\nu_{\text{pred}}$ without transfer learning are 0.193% and 0.539%, while those with transfer learning are 0.193% and 0.139%, respectively. In the example with $E_{\text{exact}}\;=\;150$ kPa and $\nu_{\text{exact}}=0.3$, the relative errors of $E_{\text{pred}}$ and $\nu_{\text{pred}}$ without transfer learning are 0.104% and 0.139%, while those with transfer learning are 0.0459% and 4.749%, respectively. These results indicate that training the inverse problems de novo yields a more accurate estimation of the unknown parameters.

## 4 Discussion

We present a generalized approach for PINNs to solve inverse problems in solid mechanics. Traditionally, the inverse FEA is a popular choice for solving inverse problems. However, the convergence of inverse FEA is highly dependent on the mesh quality and measured data. Although the application of PINNs for solving the inverse problem in solid mechanics is still in its infancy, the neural network approach for inverse problems has been demonstrated to have advantages, such as its insensitivity to noisy and incomplete data^[56]^. A rigorous comparison of prediction accuracy against the literature is infeasible, because we could not find similar application examples in the literature. As a rough comparison against the previous studies, Haghighat et al.^[27]^ reported relative material parameter approximation errors in the range of 5.86% in a von Mises elastoplasticity problem, and Zhang et al.^[46]^ reported a relative error in the range of 3% to 13.9% when estimating the shear modulus of a soft circular inclusion embedded within a square domain. Our method is able to successfully identify the unknown material parameters within 1% of relative errors in 4 out of 5 test examples, and within 2.5% in all examples. This suggests that our novel approach can produce highly accurate estimations of material parameters in the linear elastic and hyperelastic domains in both steady-state and dynamic situations. As such, PINN has substantial potential for application in diverse fields dependent upon solid mechanics and biomechanics. The excellent prediction accuracy in our work indicates a promising framework for improving engineering system performance and material designs.

Although publications on PINNs have grown exponentially since the publication by Raissi et al.^[26]^ in 2019, the previous work on applying PINNs for solving inverse problems in continuum solid mechanics is sparse, especially for 2D or three-dimensional (3D) problems. Researchers recently have applied the weak form of conservation equations to identify material properties[^57^]. However, the weak form requires a denser mesh than the strong form to obtain accurate integral estimations and parameter predictions. This limitation will result in higher computational costs as the complexity of the problem increases. At the time of writing, there are only a few research articles that describe a framework for material identification in the linear elastic and hyperelastic domains, utilizing the strong form of the conservation lawst^[27,46]^. This is partially due to the challenges of obtaining satisfactory parameter estimations for inverse problems in most practical engineering applications. In many realistic solid mechanics problems, the mechanical quantities (i.e., the displacement and stress fields) as well as the material constants (e.g., Young’s modulus E and Poisson’s ratio ν) are often differed by multiple orders of magnitude. The displacement fields are commonly tiny, in the present work ranging from $O(10^{-1})m$ to $O(10^{-3})m$, compared with the magnitude of the stress fields, in the present work ranging from O(1)Pa to $O(10^{2})\;\text{Pa}$. With such a small displacement magnitude, the loss term ${ℒ}_{\text{Data}}$ for the displacement fields becomes insensitive to the deviations of network parameters, $\theta_{\text{NN}}$. In addition, the magnitude of the material parameter E is commonly many orders higher than the material parameter ν. This huge disparity between E and ν presents difficulties in identifying an accurate solution for ν as E dominates the mechanical response mathematically. Because of the preceding reasons, a pre-trained network is used to estimate the unknown parameters of interest in Refs. [27] and [46].

Using a pre-trained network may reduce training time for problems of similar variants. Otherwise, it may be less helpful. The inversion examples in the present work are performed de novo without reliance on a pre-trained network; this highlights the inherent generalizability of our framework. We mitigate the challenges described above through the following steps. First, we use an independent network for each output variable, as suggested in Ref. [27]. Second, we determine appropriate observation point sampling strategies for the problems of interest to ensure that the influence of all material parameters is captured (as demonstrated in the cantilever beam example, the Poisson’s effect is insensitive in the regions away from the fixed boundary, which contributes to the difficulties of approximating the unknown parameter ν). Third, we reduce the weight of PDE loss to increase the influence of data in the training process. Fourth, we transform each network output variable by multiplying its corresponding maximum absolute value in reference data and apply hard constraints if needed. In our experience, the previous four steps are sufficient for identifying the material parameters in most cases. In situations where the network fails to converge due to considerable differences between E and ν, for example, in our 2D linear elastic dynamic case, we reduce the magnitude order of E in the approximation process to improve the influence of ν in the estimated stress fields. In linear elastic problems, the material parameter E and the displacement fields are inversely proportional. That means, reducing the magnitude order of E will increase the same magnitude order in the predicted displacement fields. As such, appropriate transformation of the predicted displacement fields is applied. All simulations are performed on an NVIDIA A100-SXM4-40GB GPU with an Intel Xeon CPU E5-2680 v3 computing node. The training time for the time-dependent longitudinal vibration (100 thousand iterations) and time-dependent lateral vibration (1 million iterations) is around 1.3 min and 26 min, respectively. The training time of the linear elastic steady-state (1 million iterations), hyperelastic steady-state (1 million iterations), and linear elastic dynamic (1.5 million iterations) examples is around 2.2 h, 2.5 h, and 5.8 h, respectively. In our examples, transfer learning does not offer substantial improvements in accuracy and computational time.

Further, PINNs hold several benefits over traditional engineering methods. Traditional numerical methods, such as finite element and finite volume, typically rely on complex spatial and temporal discretization schemes that could easily result in thousands of lines of code. In addition, the solution accuracy in classic numerical methods strongly depends on the mesh quality and element formulation. Numerical solutions from mesh-based finite element and finite volume methods are highly susceptible to numerical instability when handling complex geometry due to element distortion. Unlike classic mesh-based methods, PINNs are mesh-free, which eliminates element-related challenges. In addition, PINNs work directly with the strong form of conservation equations. The partial derivatives in the governing equation are computed using automatic differentiation, bypassing the need for numerical discretization schemes. Furthermore, the development of high-level deep learning libraries such as DeepXDE^[25]^ has allowed PINN frameworks to be easily set up in less than one hundred lines of code. These simple-to-use and user-friendly libraries drastically reduce the time needed to build and apply algorithms for inverse analyses. Finally, unlike traditional numerical methods, in which the parameter search process starts from scratch in every new inverse analysis, the PINN network parameters can be stored and reused when solving similar problems to improve network training time and solution accuracy.

Potential applications for determining material properties using PINNs are diverse. Computational modeling of the physical behavior of biological tissues has significant potential to inform patient-specific medicine^[58-60]^. For example, the in silico modeling of cardiac valves has the potential to allow the optimization of valve repair techniques before actual application of the repair in a patient^[61-63]^. However, accurate results will depend on knowledge of the material properties of the valve leaflets, which may vary across age, valve type, and specific pathology. The capability of PINNs to determine physical parameters even in the setting of missing or noisy data makes it well suited to extract material properties from clinical medical imaging (3D ultrasound, computed tomography, magnetic resonance imaging) of individual patients, which in turn facilitates the application of precision medicine based on computational models. This example generalizes to many applications common in biological systems. The combination of sparse data and a physical framework can be leveraged to answer questions where traditional approaches may not be feasible or even capable of generating a solution.

## 5 Conclusions

We describe the development and use of PINNs to identify the unknown material parameters in five classic solid mechanic examples. We compare the solution accuracy of soft and hard constraint formulation, as well as explore the optimal observation sampling point strategies. Further, we study the effects of transfer learning on solving inverse problems. We achieve solution accuracy within 2.5% in all examples. This work provides proof of concept that our PINN framework will work for material parameter estimation.

In the examples, it is found that the optimal choice of hard boundary auxiliary functions is problem-dependent. In this study, we select the auxiliary functions by trial and error to achieve better accuracy. In the future, we will develop an automatic approach for identifying the optimum auxiliary function. With a robust PINN framework, we plan to extend the application of PINNs in multiple domains, such as complex biological systems in medicine.

## Figures and Tables

**Table 1:** Relative errors of the unknown parameters, $E_{\text{pred}}$ and $\nu_{\text{pred}}$, and the resulting displacements and stress fields. The relative errors of both the estimated material constants and mechanical quantities are well under 1% for the steady-state examples. For the dynamic example, the relative errors of the material parameters and mechanical quantities are under 2.5% and 4%, respectively

Case	Relative error/%						
	$E_{\text{pred}}$	$\nu_{\text{pred}}$	$u_{x}$	$u_{y}$	$\sigma_{xx}$	$\sigma_{yy}$	$\sigma_{xy}$
2D steady state, linear elastic case							
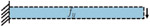	0.047	0.539	0.049	0.048	0.018	0.553	0.209
2D steady state, hyperelastic case							
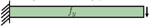	0.046	0.059	0.034	0.034	0.004	0.087	0.038
2D dynamic, linear elastic case							
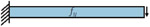	2.031	1.377	3.371	3.278	3.084	3.389	3.832

**Fig. A1:**
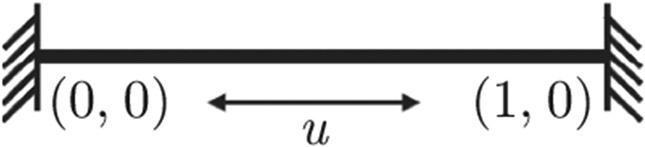
Longitudinal vibration. The beam is fixed on both ends. An initial longitudinal vibration, u(x,0)=sin(πx), is applied to the beam

**Fig. A2:**
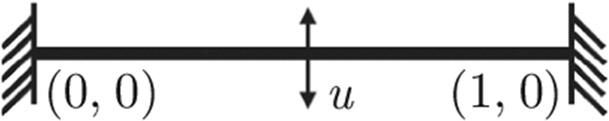
Lateral vibration. The beam of length L=1 is fixed on both ends. We apply an initial lateral vibration, u(x,0)=sin(πx), to inject lateral vibration to the system

**Fig. A3:**
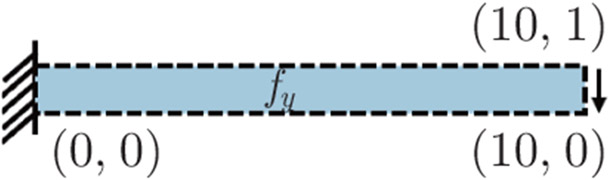
2D linear elastic cantilever beam, whose dimension is 10m × 1m and is subject to a downward body force $f_{y}=1\;N$

**Fig. A4:**
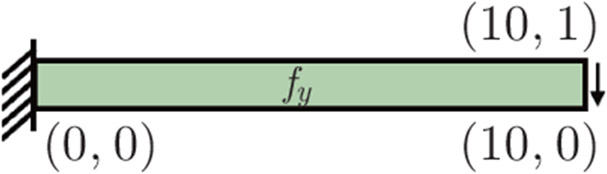
2D hyperelastic cantilever beam, whose dimension is 10m × 1m and is subject to a body force $f_{y}=0.1\;N$

**Fig. C1:**
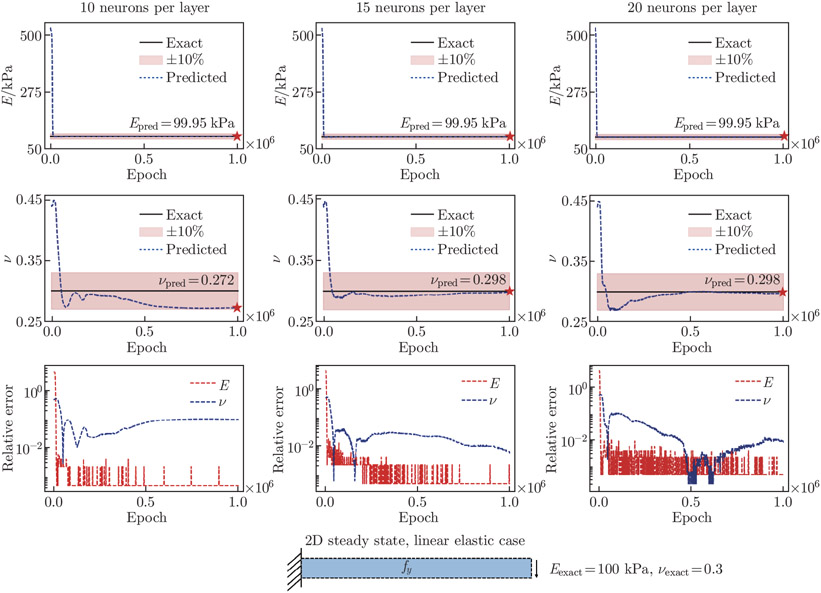
Parametric study of the network size on prediction accuracy. We compare the relative errors of $E_{\text{pred}}$ and $\nu_{\text{pred}}$ for three different network sizes. In the first network architecture (5-3-10), the relative errors of $E_{\text{pred}}$ and $\nu_{\text{pred}}$ are 0.047% and 7.04%, respectively. In the second network architecture (5-3-15), the relative errors of $E_{\text{pred}}$ and $\nu_{\text{pred}}$ are 0.047% and 0.18%, respectively. In the third network architecture (5-3-20), the relative errors of $E_{\text{pred}}$ and $\nu_{\text{pred}}$ are 0.21% and 2.56%, respectively

## Appendix A Additional details on the test examples

### A1 1D longitudinal vibration

In the longitudinal vibration example, a beam with the length L=1 is subject to an initial longitudinal vibration u, as shown in Fig. A1.

The mathematical model of this example can be characterized by the wave equation,

$$\frac{\partial^{2}u}{\partial t^{2}}=\alpha^{2}\frac{\partial^{2}u}{\partial x^{2}}$$

with the following BCs and ICs:

$$u(0,t)=u(1,t)=0,\;\;\text{for}\;\;0\leq t\leq 1,\;u(x,0)=\sin(\pi x),\;\;\frac{\partial u(x,0)}{\partial t}=0\;\;\text{for}\;\;0\leq x\leq 1.$$

The unknown variable α is set to 1. The analytical solution of this example is

$$u^{*}=\sin(\pi x)\;\cos(\pi t).$$

In this example, the weights in the loss function $w_{i}$ are set to 1, where i can be ICs, BCs, PDEs, and data.

### A2 1D lateral vibration

In the second example, an Euler-Bernoulli beam with the length L=1 is subject to an initial lateral displacement u, as shown in Fig. A2.

The Euler-Bernoulli beam equation of the lateral vibration system shown above is expressed as

(A1)
$$\frac{\partial^{2}u}{\partial t^{2}}=-\alpha^{2}\frac{\partial^{4}u}{\partial x^{4}}$$

with the following BCs and ICs:

$$u(0,t)=u(1,t)=0\;\;\text{for}\;\;0\leq t\leq 1,\;u(x,0)=\sin(\pi x),\;\;\frac{\partial u(x,0)}{\partial t}=0\;\;\text{for}\;\;0\leq x\leq 1,\;\frac{\partial^{2}u(0,t)}{\partial x^{2}}=\frac{\partial^{2}u(0,t)}{\partial x^{2}}=0\;\;\text{for}\;\;0\leq t\leq 1.$$

Similar to the 1D lateral vibration example, we set α to 1. The analytical solution of Eq. (A1) becomes

$$u^{*}=\sin(\pi x)\;\cos(\pi^{2}t).$$

In this example, the weights in the loss function $w_{i}$ are also set to 1, where i can be ICs, BCs, PDEs, and data.

### A3 2D linear elastic cantilever beam

In the third example, we consider a 10m × 1m cantilever beam fixed on the left end, as shown in Fig. A3. The beam is made of linear elastic material and is subject to a downward body force $f_{y}=1\;N$.

In this steady-state example, we assume plane-stress formulation. The momentum balance equation is expressed as

$$\sigma_{ij,j}+f_{i}=0.$$

The isotropic linear elastic material constitutive model is defined as

σ=C⋅ϵ

with

$$\sigma =\left(\begin{array}{c} \sigma_{xx}\\ \sigma_{yy}\\ \sigma_{xy} \end{array}\right),\;\;C=\frac{E}{\left(1-\nu^{2}\right)}\left(\begin{array}{ccc} 1 & \nu & 0\\ \nu & 1 & 0\\ 0 & 0 & \left(1-\nu \right) \end{array}\right),\;\;\epsilon =\left(\begin{array}{c} \epsilon_{xx}\\ \epsilon_{yy}\\ \epsilon_{xy} \end{array}\right).$$

The kinematic relation is

$$\epsilon_{xx}=\frac{\partial u_{x}}{\partial x},\;\;\epsilon_{yy}=\frac{\partial u_{y}}{\partial y},\;\;\epsilon_{xy}=\frac{1}{2}\left(\frac{\partial u_{x}}{\partial y}+\frac{\partial u_{y}}{\partial x}\right).$$

Young’s modulus and Poisson’s ratio of this example are 10^5^ Pa and 0.3, respectively. The weights in the loss function $w_{\text{PDEs}}$ and $w_{\text{Data}}$ are set to 10^−10^ and 1, respectively.

### A4 2D hyperelastic cantilever beam

In the fourth example shown in Fig. A4, we consider the same geometry as the third example but with Neo-Hookean material.

The constitutive model for compressible isotropic hyperelastic material is expressed as

$$\sigma_{ij}=\frac{1}{J}P_{ik}F_{kj}^{T},$$

where F is the deformation gradient tensor defined as $F_{ij}=\delta_{ij}+u_{i,j}$, J=detF, and P is the first Piola-Kirchhoff stress tensor. The first Piola-Kirchhoff stress for compressible Neo-Hookean material is as follows:

$$P=\frac{\partial \Psi }{\partial F}=\mu F+(\lambda \;\text{ln}\;J-\mu )F^{-T},$$

where λ and μ, are Lamé’s elasticity parameters. Further, Ψ is the strain energy density function. Assume that the plane-strain formulations are

$$\lambda =\frac{E\nu }{\left(1+\nu )(1-2\nu \right)},\;\;\mu =\frac{E}{2(1+\nu )}.$$

The Neo-Hookean strain energy density function Ψ is expressed as

$$\Psi (I_{1},J)=\frac{1}{2}\lambda (\text{ln}\;J{)}^{2}-\mu \;\text{ln}\;J+\frac{1}{2}\mu (I_{1}-2),$$

where $I_{1}=\text{tr}(F^{T}\cdot F)$. The weights in the loss function $w_{\text{PDEs}}$ and $w_{\text{Data}}$ are set to 10^−8^ and 1, respectively.

### A5 2D dynamic cantilever beam

In the fifth example, we extend the 2D linear elastic cantilever beam example to dynamic analysis. The beam has the density (kg · m^−3^). Young’s modulus and Poisson’s ratio are 10^6^ Pa and 0.3, respectively. The applied body force in this example is $f_{y}=5\;N$. The momentum balance equation becomes

$$\sigma_{ij,j}+f_{i}=\rho \partial_{tt}u_{i}.$$

Consider the plane-strain formulations. Then, the C matrix in the constitutive relation σ=C⋅ϵ is

$$C=\frac{E}{\left(1+\nu )(1-2\nu \right)}\left(\begin{array}{ccc} \left(1-\nu \right) & \nu & 0\\ \nu & \left(1-\nu \right) & 0\\ 0 & 0 & \left(1-2\nu \right) \end{array}\right).$$

The weights in the loss function $w_{\text{PDEs}}$ and $w_{\text{Data}}$ are set to 10^−8^ and 1, respectively.

## Appendix B FEA

We use FEniCS to generate the displacement and stress reference data for the 2D examples. The cantilever beam geometry is discretized into 1 000 second-order rectangular elements with Δx=Δy=0.1m. For the linear elastic static example, we validate the maximal deflection obtained from FEA against the analytical solution from the Euler-Bernoulli beam theory: $u_{y,\max}=\frac{\rho gL^{4}}{EI}$. The maximum deflections from the analytical solution and FEA are −0.15 m and −0.151 m, respectively. The relative error is approximately 0.67%. In the 2D dynamic example, a Newmark implicit time integration scheme is used to facilitate the evolution of displacement and stress in time. We validate the frequency of the y-tip displacement against the first natural frequency for the cantilever beam derived from the Euler-Bernoulli beam theory, i.e., $f=\frac{1.875^{2}}{2\pi L^{2}}\sqrt{\frac{EI}{\rho A}}$. The natural frequency from the analytical solution is 1.61 Hz, and the natural frequency from the FEA solution is 1.66 Hz. The relative error is approximately 3%.

## Appendix C Variation of network architectures

In this section, we study the influence of network architectures on PINNs’ predictive capability. In our work, we use 5 independent networks to estimate $E_{\text{pred}}$ and $\nu_{\text{pred}}$ in the 2D examples. Each independent network has 3 layers of neurons; the same numbers of neurons are applied to each layer. Here, we vary the width of the network by adjusting the numbers of neurons to 10, 15, and 20 neurons per layer. We abbreviate the three network architectures to 5-3-10, 5-3-15, and 5-3-20, wherein the first number refers to the number of independent networks, the second number refers to the number of layers per network, and the last number refers to the number of neurons per layer. The resulting $E_{\text{pred}}$ and $\nu_{\text{pred}}$ are presented in Fig. C1. As shown, the width of the neural network does not have a significant effect on the convergence of $E_{\text{pred}}$; $E_{\text{pred}}$ converges rapidly to the ground truth in less than 1 × 10^5^ epochs. As we increase the number of neurons from 10 to 15, the relative error reduces from 7.04% to 0.18%. However, $\nu_{\text{pred}}$ starts to deviate from the ground truth as we keep increasing the width of the network to 20 neurons per layer. This indicates that a network width of 15 is most suitable for the examples at hand.
